# Global Gene Expression Profiling through the Complete Life Cycle of *Trypanosoma vivax*

**DOI:** 10.1371/journal.pntd.0003975

**Published:** 2015-08-12

**Authors:** Andrew P. Jackson, Sophie Goyard, Dong Xia, Bernardo J. Foth, Mandy Sanders, Jonathan M. Wastling, Paola Minoprio, Matthew Berriman

**Affiliations:** 1 Department of Infection Biology, Institute of Infection and Global Health, University of Liverpool, Liverpool, United Kingdom; 2 Department of Infection and Epidemiology, Institut Pasteur, Paris, France; 3 Pathogen Genomics Group, Wellcome Trust Sanger Institute, Wellcome Trust Genome Campus, Hinxton, Cambridge, United Kingdom; Institute of Tropical Medicine, BELGIUM

## Abstract

The parasitic flagellate *Trypanosoma vivax* is a cause of animal trypanosomiasis across Africa and South America. The parasite has a digenetic life cycle, passing between mammalian hosts and insect vectors, and a series of developmental forms adapted to each life cycle stage. Each point in the life cycle presents radically different challenges to parasite metabolism and physiology and distinct host interactions requiring remodeling of the parasite cell surface. Transcriptomic and proteomic studies of the related parasites *T*. *brucei* and *T*. *congolense* have shown how gene expression is regulated during their development. New methods for *in vitro* culture of the *T*. *vivax* insect stages have allowed us to describe global gene expression throughout the complete *T*. *vivax* life cycle for the first time. We combined transcriptomic and proteomic analysis of each life stage using RNA-seq and mass spectrometry respectively, to identify genes with patterns of preferential transcription or expression. While *T*. *vivax* conforms to a pattern of highly conserved gene expression found in other African trypanosomes, (e.g. developmental regulation of energy metabolism, restricted expression of a dominant variant antigen, and expression of ‘Fam50’ proteins in the insect mouthparts), we identified significant differences in gene expression affecting metabolism in the fly and a suite of *T*. *vivax*-specific genes with predicted cell-surface expression that are preferentially expressed in the mammal (‘Fam29, 30, 42’) or the vector (‘Fam34, 35, 43’). *T*. *vivax* differs significantly from other African trypanosomes in the developmentally-regulated proteins likely to be expressed on its cell surface and thus, in the structure of the host-parasite interface. These unique features may yet explain the species differences in life cycle and could, in the form of bloodstream-stage proteins that do not undergo antigenic variation, provide targets for therapy.

## Author Summary

*Trypanosoma vivax* is a single-celled parasite that infects cattle and non-domesticated animals through the bite of the tsetse fly. The parasite causes animal trypanosomiasis, a chronic condition resulting in severe anemia, muscle wastage and ultimately death if untreated. This disease is endemic across sub-Saharan Africa but has also spread to South America and causes considerable losses in animal productivity, impeding economic development in the world’s poorest nations. To develop new ways of preventing and treating animal trypanosomiasis, we need an accurate understanding of how the parasite causes disease. In this study, we present an analysis of gene expression throughout the *T*. *vivax* life cycle that compares the abundance of gene transcripts (mRNA) and proteins in the mammalian and insect hosts. We have identified genes that are preferentially expressed in each life stage, including many that are unique to *T*. *vivax* and probably expressed on its cell surface. Our findings provide a comprehensive understanding of how gene expression is regulated in *T*. *vivax* and further refine a pool of *T*. *vivax*-specific genes that could be exploited to prevent and treat animal trypanosomiasis.

## Introduction

African trypanosomes are unicellular vector-borne hemoparasites of humans, domestic livestock and wild animals. They cause African trypanosomiasis, an endemic disease of sub-Saharan Africa otherwise known as sleeping sickness in humans and nagana in animals, and are transmitted between vertebrate hosts by the bite of tsetse flies (*Glossina* spp.). This endemic disease causes considerable morbidity in livestock herds and associated losses in animal productivity. The threat of Animal African trypanosomiasis in tsetse-infested areas also prevents effective exploitation of available pasture, thereby impeding economic development in the world’s poorest nations.

**Fig 1:**
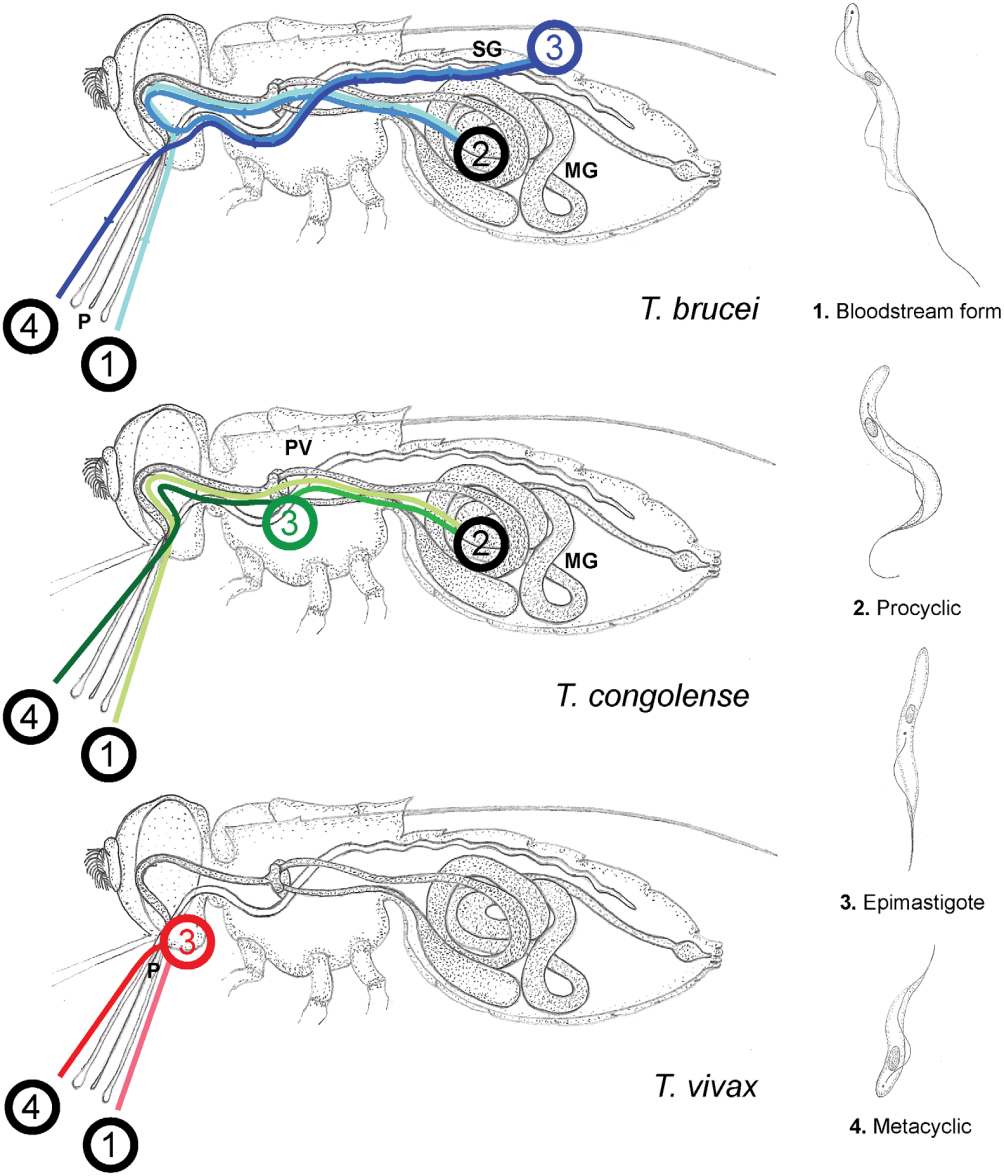
Life cycle variation among African trypanosomes. The passage and development of African trypanosomes through the fly vector varies by species. The figure shows the route taken by each species through the vector and position of each developmental stage. Insect anatomy is abbreviated thus: proboscis (P), mid-gut (MG), salivary gland (SG) and proventriculus (PV). The life cycle within the fly is defined here by four stages: (1) Ingestion of bloodstream-form parasites; (2) migration of parasites to the insect mid-gut with differentiation into procyclic forms (*T*. *vivax* lacks this stage); (3) migration anteriorly to the proboscis (*T*. *vivax*), proventriculus (*T*. *congolense*) or salivary gland (*T*. *brucei*) and differentiation into epimastigote forms; and (4) differentiation into metacyclic forms and inoculation into the vertebrate host upon insect feeding.

There are several species of African trypanosome that vary in life cycle, host range and pathology. *Trypanosoma brucei* is predominantly an animal pathogen that has evolved the ability to infect humans on multiple occasions [1], while *T*. *congolense* and *T*. *vivax* are exclusively animal pathogens. During their life cycles, *T*. *brucei* and *T*. *congolense* exist as procyclic forms in the mid-gut of the tsetse fly before migrating into the salivary glands and proventriculus respectively, where they develop into epimastigotes and then metacyclic trypomastigotes that are able to infect vertebrates (see Fig 1). In contrast, *T*. *vivax* lacks a procyclic stage in the insect mid-gut and has no complex migration within the insect; rather, *T*. *vivax* develops directly into epimastigote forms within the insect proboscis [2] (Fig 1). This difference might explain why *T*. *vivax* can be transmitted by other kinds of biting insect [3–4] and has therefore spread beyond the sub-Saharan distribution of the tsetse fly into northern Africa and South America [5–6].

In recent years our understanding of trypanosome biology has progressed substantially through the determination of genome sequences for *T*. *brucei* [7–10] and for *T*. *congolense* and *T*. *vivax* [11], as well as numerous analyses of gene expression, (largely confined to *T*. *brucei*, except for three studies [12–14]), using transcriptomic [15–20] and proteomic [21–23] approaches. Consequently, we now know that the complex life cycle of African trypanosomes is facilitated by considerable developmental regulation of gene expression. Developmental regulation in *T*. *brucei* is particularly apparent in the expression of major surface glycoproteins belonging to the procyclic, epimastigote and bloodstream forms respectively, i.e. procyclin [24], *B* *rucei* Alanine-Rich Protein (BARP; [25]) and the Variant Surface Glycoprotein (VSG).

There are compelling reasons for supposing that gene expression in *T*. *vivax* will be different to *T*. *brucei* in important ways, not least due to differences in life cycle development (Fig 1), but also because the *T*. *vivax* genome contains substantially different repertoires of VSG and BARP-like genes (and no procyclin at all), as well as numerous gene families that appear to be unique [26]. As *in vitro* cultivation of insect stages has not previously been possible, gene expression in *T*. *vivax* has only been analyzed in the bloodstream form, and then only through transcriptomic analysis [14]. Moreover, given that gene regulation is achieved largely through post-transcriptional modifications in trypanosomes (reviewed in [27]), differences between transcript and peptide abundances across the life cycle are expected. We recently established *in vitro* cultures of the insect stages of *T*. *vivax* [28], and so a comparison of gene expression across African trypanosome species is now possible.

Using transcriptome sequencing and proteomics, we have analyzed differences in gene expression between *T*. *vivax* epimastigote, metacyclic and bloodstream forms. Our results show that the numerous *T*. *vivax*-specific genes predicted to function on the parasite cell surface are transcribed and often developmentally regulated. Genome-wide patterns of developmental regulation are conserved across African trypanosome species, with some notable exceptions concerning pyruvate metabolism in *T*. *vivax*, which might indicate an important species difference in energy metabolism. Comparative genomics suggests that *T*. *vivax* differs quite considerably from the model *T*. *brucei*; by illuminating the expression of distinctive features in the *T*. *vivax* genome, this study moves us closer to understanding their phenotypic effects.

## Methods

### Ethics statement

All mice were housed in the Institut Pasteur animal care facilities in compliance with European animal welfare regulations (European Convention for the Protection of Vertebrate Animals used for Experimental and other Scientific Purposes CETS No.: 123). Institut Pasteur is a member of Committee #1 of the Comité Régional d’Ethique pour l’Expérimentation Animale (CREEA), Ile de France. Animal housing conditions and the protocols used in the work described herein were approved by the ‘‘Direction des Transports et de la Protection du Public, Sous-Direction de la Protection Sanitaire et de l’Environnement, Police Sanitaire des Animaux” (#B 75-15-28), in accordance with the Ethics Charter of animal experimentation that includes appropriate procedures to minimize pain and animal suffering. Authorization (to PM) to perform experiments on vertebrate animals is granted by license #75–846 issued by the Paris Department of Veterinary Services, DDSV.

### Cell culture

*Trypanosoma* (*Duttonella*) *vivax* IL 1392 was originally derived from the Zaria Y486 Nigerian isolate. Bloodstream form parasites were maintained *in vivo* by continuous passage in mice, as previously described [29]. Once parasitemia reached at least 5x10^8^ parasites per ml blood was collected by cardiac puncture onto heparin (2500 IU/kg), and was then diluted 1: 10 (v/v) with PBS 0.5% glucose to 5x10^7^ parasites per ml. Parasites were separated from red blood cells by differential centrifugation using a swing-out rotor (Jouan GR412, Fisher Bioblock Scientific, Strasbourg, France). Diluted blood was processed by one round of centrifugation (5 min at 200 g) and the supernatant withdrawn with a pipette without disturbing the red blood cell layer and the thin interface containing the white blood cells. Parasite enriched suspension was submitted to a second round of centrifugation (5 min at 200 g) to eliminate all residual cells. The supernatant was then centrifuged for 10 min at 1800 g and bloodstream form-containing pellets devoid of host cells were submitted to two further PBS washes under the same centrifugation conditions. Bloodstream form-containing pellets were further treated for RNA or protein extractions.

*T*. *vivax* epimastigote cultures have been previously described [28]. Briefly, bloodstream forms purified as described above from infected mice differentiated into epimastigotes in TV3 media: IMDM 50%, DMEM (without glucose) 10% heat-inactivated fetal calf serum (FBS, MP Biomedicals or Invitrogen) and/or 10% heat-inactivated goat serum (GS, Invitrogen), 0.03 mM bathocuproinedisulfonic acid, 0.45 mM L-cysteine, 0.2 mM hypoxanthine, 0.14 mM ß-mercaptoethanol, 4mM L-proline, 0.05 mM thymidine, and 25 mM HEPES pH7.4. All supplements were obtained from Sigma Aldrich except HEPES (Invitrogen, Cergy Pontoise). Epimastigote growth cultures were maintained *in vitro* by serial passages. Epimastigotes attached to the surface of the culture flask formed micro-colonies and covered the entire surface after two weeks; the number of cells in the supernatant increased proportionally to the density of the adherent cell layer. Adherent epimastigotes were recovered from the flask by scraping and washed three times with PBS. As previously described, metacyclic forms are produced during *in vitro* growth and are found in the cell culture supernatant [28]. Metacyclic forms were isolated from the cell culture using an approach derived from “bovine plasma aggregation method” [30]. Supernatant from a dense culture (14 days) was remove from the flask, 30% non-inactivated goat serum was added to the cells and incubated at 27°C for 30 min. During the incubation period, epimastigotes aggregate into cell clumps, while metacyclic forms continue to swim freely. The metacyclic forms were then separated from the epimastigote clumps by passing the trypanosome suspension through a 5 μm pore size filter (Millipore Cat. Bedford, MS, USA). The metacyclic forms were then concentrated and washed by centrifugation at 750g for 15 min in 14 ml conical centrifuge tubes and RNA or protein prepared from the resultant cells pellets.

### Sample preparation for RNA-seq

Total RNA was isolated using an RNeasy Mini Kit (Qiagen, Courtaboeuf, France) in accordance with the manufacturer's instructions. RNA purity and concentration were evaluated by spectrophotometry using NanoDrop ND-2000 (ThermoFisher). RNA quality and the relative contributions of total and small RNA were assessed by the Agilent 2100 Bioanalyzer microfluidics-based platform (Agilent Technologies, Santa Clara, USA). Four biological replicates were prepared for bloodstream form and metacyclic cells each. Five replicates were produced for epimastigote cells.

### RNA sequencing

For each replicate, poly-adenylated RNA (mRNA) was purified from total RNA using an oligo-dT magnetic bead pull-down, using TruSeq RNA Sample Prep v2 kits (Illumina). The mRNA was then fragmented using metal ion-catalyzed hydrolysis. A random-primed cDNA library was synthesized and double-strand cDNA was used as the input to a standard Illumina library preparation, with a fragment size of 400bp. The libraries were amplified with 10 cycles of PCR using KAPA Hifi Polymerase. Samples were quantified and pooled based on a post-PCR Agilent Bioanalyzer, followed by size-selection using the LabChip XT Caliper. The multiplexed library was sequenced on the Illumina HiSeq 2000 with forward and reverse primers, according to the manufacturers standard protocol, resulting in 100-nucleotide paired-end reads. Sequenced data was analyzed and quality controlled and individual indexed library BAM files created.

### Transcriptomic data analysis

Paired-end RNA-seq data were mapped to the *T*. *vivax* Y486 reference strain [11] (downloaded from TritrypDB release 6.0) using Bowtie2 [31] under the default parameters and within the Galaxy bioinformatics platform [32]. Transcript abundance for each replicate was estimated across the genome using Cufflinks [33] and measured in Fragments Per Kilobase Mapped (FPKM). The option for quartile normalization within Galaxy was applied to maximize our ability to detect preferential expression of low abundance transcripts against the background of highly abundant species. The option for bias detection and correction was enforced. The option for multi-read correction was applied because some of our genes of interest are multi-copy and may map to multiple locations. Fold change in transcript abundance, and significance of differential expression, was estimated using Cuffdiff [33] for three pairwise comparisons of *T*. *vivax* life stages, combining all replicates in each case. Cuffdiff applies the Benjamini-Hochberg correction for multiple testing when assessing the significance of fold changes. To ensure accurate assessment of differential expression, transcript abundance was corroborated using a second method, edgeR [34]. Correlations for fold change in transcript abundance returned by Cufflinks and edgeR displayed high congruence when comparing life stages (r^2^ = 0.89–0.91). Significant differences in transcript expression were defined as at least 2-fold enrichment between conditions and q < 0.05, where q is the p value corrected for false discovery rate (FDR).

### Sample preparation for proteomics

Protein from cell lysates was dispensed into low protein-binding microcentrifuge tubes (Sarstedt, Leicester, UK) and made up to 160 μl by addition of 25 mM ammonium bicarbonate. The proteins were denatured using 10 μl of 1% (w/v) RapiGest (Waters MS Technologies, Manchester, UK) in 25 mM ammonium bicarbonate followed by three cycles of freeze-thaw, and two cycles of 10 min sonication in a water bath. The sample was then incubated at 80°C for 10 min and reduced with 3 mM dithiothreitol (Sigma-Aldrich, Dorset, UK) at 60°C for 10 min then alkylated with 9 mM iodoacetamide (Sigma-Aldrich, Dorset, UK) at room temperature for 30 min in the dark. Proteomic grade trypsin (Sigma-Aldrich, Dorset, UK) was added at a protein:trypsin ratio of 50:1 and samples incubated at 37°C overnight. Three biological replicates were prepared for each cell type.

### LC-MS/MS analysis

Peptide mixtures were analyzed by on-line nanoflow liquid chromatography using the nanoACQUITY-nLC system (Waters MS technologies, Manchester, UK) coupled to an LTQ-Orbitrap Velos (ThermoFisher Scientific, Bremen, Germany) mass spectrometer equipped with the manufacturer’s nanospray ion source. The analytical column (nanoACQUITY UPLCT BEH130 C18 15 cm x 75 μm, 1.7 μm capillary column) was maintained at 35°C and a flow-rate of 300nl/min. The gradient consisted of 3–40% acetonitrile in 0.1% formic acid for 90 min then a ramp of 40–85% acetonitrile in 0.1% formic acid for 3 min. Full scan MS spectra (m/z range 300–2000) were acquired by the Orbitrap at a resolution of 30,000. Analysis was performed in data-dependent mode. The top 20 most intense ions from MS1 scan (full MS) were selected for tandem MS by collision induced dissociation (CID) and all product spectra were acquired in the LTQ ion trap. Ion trap and Orbitrap maximal injection times were set to 50 ms and 500 ms, respectively.

### Proteomic data analysis

Thermo RAW files were imported into Progenesis LC–MS (version 4.1, Nonlinear Dynamics, UK). Runs were time aligned using default settings and using an auto selected run as reference. Peaks were picked by the software and filtered to include only peaks with a charge state of between +2 and +6. Peptide intensities were normalized against the reference run by Progenesis LC-MS and these intensities are used to highlight differences in protein expression between control and treated samples with supporting statistical analysis (ANOVA and q-values) calculated by the Progenesis LC-MS software. Spectral data were transformed to mgf files with Progenesis LC-MS and exported for peptide identification using the Mascot (version 2.3.02, Matrix Science) search engine. Tandem MS data were searched against a custom database that contained the common contamination and protein sequences predicted for the *T*. *vivax* reference genome (downloaded from TriTrypDB v-6.0). Search parameters were as follows; precursor mass tolerance set to 10ppm and fragment mass tolerance set to 0.5 Da. One missed tryptic cleavage was permitted. Carbamidomethylation (cysteine) was set as a fixed modification and oxidation (methionine) set as a variable modification. Mascot search results were further processed using the machine learning algorithm Percolator. The false discovery rates were set at 1% and at least two unique peptides were required for reporting protein identifications. Protein abundance (iBAQ) was calculated as the sum of all the peak intensities (from Progenesis output) divided by the number of theoretically observable tryptic peptides [35]. Protein abundance was normalized by dividing the protein iBAQ value by the summed iBAQ values for that sample. The reported abundance is the mean of the biological replicates.

### Data accessibility

All cDNA sequence data are available from the European Nucleotide Archive (http://www.ebi.ac.uk/ena), accession number ERP001753. Details of the transcriptomic experiments are also available from the Array Express website (https://www.ebi.ac.uk/arrayexpress/), accession number E-ERAD-100. The mass spectrometry proteomics data have been deposited with the ProteomeXchange Consortium via the PRIDE partner repository (http://www.ebi.ac.uk/pride/archive/) with the dataset identifier PXD001617.

## Results

### Estimation of transcript and peptide abundance

By exploiting new protocols for the *in vitro* cultivation of *T*. *vivax* epimastigote and metacyclic forms, we have produced comparative transcriptomic and proteomic data for the whole *T*. *vivax* life cycle using RNAseq and LC-MS/MS approaches respectively. Transcripts were detected for 10116 *T*. *vivax* Y486 genes (85.1% of all genes); 8994 of these transcripts (88.9%) were observed with at least 10 FPKM. The abundance of each transcript, as estimated using Cufflinks [33], is described in S1 Table. The most abundant transcripts in the bloodstream form were derived from tubulins, diverse ribosomal proteins and VSG-like sequences TvY486_0009580 (17331 FPKM) and TvY486_0018880 (9669 FPKM), which are assumed to have been the active VSG at the time of sequencing. Besides these, abundant transcripts encoding named proteins concern glyceraldehyde 3-phosphate dehydrogenase (TvY486_0603710; 1512 FPKM), a receptor-type adenylate cyclase (TvY486_0029610; 1210 FPKM), cathepsin B-like cysteine peptidase (TvY486_0600060; 794 FPKM), and an uncharacterized gene specific to *T*. *vivax* (TvY486_0900440; 1646 FPKM). The most abundant transcripts in the epimastigote and metacyclic cells encoded the same set of highly abundant tubulins and ribosomal proteins, but not the putative VSG, displaying instead an abundance of BARP-like proteins TvY486_0012620 (975 FPKM) and TvY486_1114940 (847 FPKM). Abundance estimates across our independent replicates were consistent, with strong positive correlations of replicates (ranging from 0.94 to 0.99) across all life stages (S1 Fig); and when fold change in transcript abundance is compared between life stages using edgeR, replicates cluster by stage illustrating their consistency (S2 Fig).

Peptide abundance, as defined by quantitative analysis with MASCOT, is described in S2 Table. 11099 peptides were counted corresponding to 1952 proteins (16.3% of predicted proteome). Of these, 1245 were sufficiently abundant to be quantified by iBAQ (i.e. two unique peptides were observed with a FDR of 0.01). Of these, 798 had a q value < 0.05, meaning that differential expression can be reliably inferred. The most abundant peptides in the bloodstream form were alpha and beta tubulin, various histones, putative VSG (TvY486_0009580/TvY486_0018880; i.e. coinciding with the most abundant VSG-like transcripts), and metabolic enzymes such as fructose-bisphosphate aldolase, enolase, glutamate dehydrogenase, arginine kinase, phosphoglycerate mutase, succinyl-coA:3-ketoacid-coenzyme A transferase and glycerol-3-phosphate dehydrogenase. The most abundant peptides in the epimastigote and metacyclic stages largely belonged to the same set of proteins, except that VSG were not observed and glycolytic enzymes were less abundant. As with transcript abundance, the proteome was consistent between independent replicates for each life stage, as illustrated in a principle component analysis in which replicates cluster tightly by stage (S3 Fig).

The degree to which relative abundance of transcripts and peptides concur throughout the life cycle is an important question with implications for regulation of gene expression, especially in trypanosomatids in which regulation is thought to be mostly post-transcriptional [27]. Correlations of transcript and peptide abundance across the genome (a-c) and for differentially expressed genes in each life stage (d-f) are shown in S4 Fig. These graphs show that the correlation is poor for all genes (r^2^ between 0.22 and 0.36) but improved for genes with evidence of developmental regulation (r^2^ between 0.38 and 0.65).

### Developmental regulation of transcripts

Differential expression is defined as significant where transcript abundance displays at least two-fold enrichment and where q < 0.05. We found that 11.2% (1137) of transcripts showed significant differential expression in one or more stage comparison; we refer to these as ‘developmentally regulated’ and they are listed in S3 Table. In bloodstream forms, 518 transcripts were significantly more abundant relative to epimastigotes, and 382 transcripts were significantly more abundant in bloodstream forms relative to metacyclics. The greatest enrichment in favor of bloodstream forms concerned the putative active VSG (TvY486_0018880; fold-change (FC) = 110.9); other large fold-changes that implicated named sequences concerned three receptor-type adenylate cyclases (TvY486_0026190, TvY486_0003180 and TvY486_0029610; FC = 49.8, 18.9 and 10.8 respectively), a glycerol-3-phosphate dehydrogenase (TvY486_0802930; FC = 9.4) and a phospholipase A1 (TvY486_0102170; FC = 15.6). Besides these instances, the majority of transcripts (83%) preferentially expressed in bloodstream forms encode hypothetical proteins. Among these are hypothetical proteins belonging to *T*. *vivax*-specific families that are included in a Cell-Surface Phylome (CSP) that we published previously [26] for gene families predicted to be expressed on the cell surfaces of the three principal African trypanosome species; i.e. Fam30 (e.g. TvY486_0003670; FC = 29.4), Fam28 (e.g. TvY486_0030920; FC = 27.5), Fam34 (e.g. TvY486_0009950; FC = 26.5) and Fam31 (e.g. TvY486_0000210; FC = 23.4). Yet another family of uncharacterized genes, unique to *T*. *vivax* but not included in the CSP presently, show greater differential expression in bloodstream forms than any other family except for VSG. This gene family occurs 25 times among transcripts up-regulated in bloodstream forms relative to epimastigotes (S3 Table) and provides four of the 20 largest fold-changes in favor of bloodstream forms (e.g. TvY486_0033680, FC = 38.1). A BLASTp analysis shows that this family has at least 44 members across the *T*. *vivax* genome but none of these paralogs were observed to be preferentially expressed in either epimastigote or metacyclic form.

In epimastigotes, we identified 393 transcripts that were developmentally regulated, 387 of which are significantly more abundant in epimastigotes relative to bloodstream forms, while 8 transcripts are significantly more abundant in epimastigotes relative to metacyclic forms (see S3 Table). The dearth of preferential expression in epimastigotes relative to metacyclics was only slightly relieved by analysis with edgeR, which reported 23 cases. Since it was necessary to grow epimastigote cultures to high density in order to achieve a high proportion of metacyclic cells, it is possible that the lack of significant differences between these cell types is due to the effects of high density on growth. As with bloodstream forms, most developmentally regulated transcripts encode hypothetical proteins (65.1%), including those with the greatest fold changes in expression, i.e. TvY486_1110640 (FC = 73.1). Transcripts preferentially expressed in epimastigotes also concern further *T*. *vivax*-specific, CSP gene families, namely Fam35 (11 paralogs; FC = 5.4–43.7) and Fam43 (three paralogs; FC = 35.0–53.8). However, these gene families were more abundant still in metacyclic forms (see below), indicating that their main focus of expression was not the epimastigote. Aside from these uncharacterized gene families, transcripts implicated in cellular respiration were also seen, for example, components of the electron transfer chain such as cytochrome c1 (TvY486_0801280; FC = 13.0), cytochrome c (TvY486_0804690; FC = 13.2) and cytochrome c oxidase subunits (FC between 4.3–20.9). Also transcripts for multiple cation transporters (FC between 3.8–24.7) and a meiotic recombination protein DMC1 (TvY486_0904120; FC = 10.5).

In the final, metacyclic life stage, 357 transcripts were significantly more abundant relative to bloodstream forms, and these gave a very similar picture to the enriched transcripts in epimastigotes. A further 136 transcripts were significantly more abundant relative to epimastigotes (see S3 Table) including the *T*. *vivax*-specific, CSP families 34, 35 and 43 (see above), and other transcripts encoding DNA polymerase kappa (TvY486_1109280; FC = 2.8), an adenylate cyclase (TvY486_0029610; FC = 2.5), and various reverse transcriptases derived from SLACS elements (average FC = 6.8).

With respect to these observations, it should be noted that further analysis using edgeR produced very similar results to Cufflinks, with only 8.6% of the gene set displaying significant differential expression. Also, there was substantial overlap in the identities of developmentally regulated transcripts between comparisons; thus, of 518 transcripts significantly enriched in bloodstream forms relative to epimastigotes, 372 of these were also enriched relative to metacyclics; similarly, of 387 transcripts significantly enriched in epimastigotes relative to bloodstream forms, 285 of these were also enriched in metacyclics relative to the latter. From this it should be clear that, of the three life stages, the epimastigotes and metacyclic transcriptomes were most alike.

We examined the developmental regulated transcripts for Gene Ontology (GO) terms that were significantly enriched using a Fishers Exact test in BLAST2GO [36]. This confirmed that transcripts preferentially expressed in bloodstream forms are enriched for terms associated with *glycolysis* (GO:0006096; q = 3.38e^-04^) and *glycosome* (GO:0020015; p = 1.83e^-03^), while those preferentially expressed in epimastigotes are enriched for *cytochrome-c oxidase activity* (GO:0004129; p = 2.01e^-13^) and *ATP synthesis coupled proton transport* (GO:0015986; p = 1.43e^-03^). While suggestive of consistent differences in energy metabolism between life stages, differences in transcript abundance do not guarantee disparity in protein expression; thus, we sought to corroborate these observations with our proteomic data.

### Developmental regulation of proteins

A protein shows significant differential expression if a constitutive peptide displays at least 2-fold enrichment and q < 0.05. Under these criteria, 595 or 30.5% of observed proteins (74.6% of quantifiable peptides) were developmentally regulated and these are listed in S4 Table. In the bloodstream form, 131 proteins were significantly more abundant relative to epimastigotes, while 63 proteins were significantly more abundant relative to metacyclics. Compared to differentially expressed transcripts, there is a smaller proportion of hypothetical proteins (29%), suggesting that our proteomic analysis has captured the most abundant components of cellular physiology that are relatively well understood. Accordingly, various structural proteins and diverse enzymes are listed, most notably components of the glycolytic pathway such as phosphoglycerate mutase (TvY486_0302920; FC = 6.6), fructose-bisphosphate aldolase (TvY486_1005670; FC = 3.5) and glycerol-3-phosphate dehydrogenase (TvY486_0802930; FC = 5.5). Analysis of functional terms associated with peptides preferentially expressed in bloodstream forms shows that *glucose metabolism* is significantly enriched (GO:0006006; FDR = 5.6e^-05^) relative to epimastigotes, while the *citric acid cycle* is significantly enriched (KEGG; FDR = 4.6e^-04^) relative to metacyclics.

The multi-copy, *T*. *vivax*-specific transcripts described above as being the most highly abundant family in bloodstream forms were also identified among our peptides. Four members of this family are preferentially expressed in bloodstream forms, one relative to epimastigotes (TvY486_0008730; FC = 15.9) and three more relative to metacyclics. None were expressed in either insect stage, further indicating that this is a novel and very prominent feature of bloodstream forms.

In the epimastigote form, 147 and 104 proteins were significantly more abundant relative to bloodstream forms and metacyclics respectively. GO terms associated with *oxidation-reduction processes* (GO:0055114; FDR = 1.1e^-03^) and *amino acid metabolism* (GO:0006520; FDR = 2.3e^-02^) were found to be significantly enriched. Peptides suggestive of oxidative phosphorylation, although no other elements of mitochondrial energy metabolism, were also significantly more abundant relative to metacyclics.

In the metacyclic stage, 62 peptides were significantly more abundant relative to bloodstream forms, and 89 peptides were significantly more abundant relative to epimastigotes. In both cases, the greatest fold changes pertain to hypothetical proteins encoded by members of CSP gene families (see below). Among the 88 peptides, various proteins with roles in intracellular trafficking were implicated, for example, the vesicle formation protein *Sec24C* (TvY486_0300580; FC = 11.5; [37]) and signal recognition particle receptor (TvY486_1110560; FC = 4.9), as well as several dynein heavy chains (FC between 2.2 and 7.1). A Fishers Exact test shows that functions associated with *intracellular transport* (GO:0046907; FDR = 2.0e^-02^) are significantly enriched. The same test shows that *purine ribonucleotide catabolism* (GO:0009154; FDR = 2.1e^-03^), (which refers here to the ATP-binding requirements of the same intracellular transport processes), is also significantly enriched.

It should be noted that, unlike the cohorts of stage-specific transcripts, there was no overlap in the membership of these various stage-specific peptide groups, i.e. there were no peptides significantly enriched in both epimastigotes and metacyclics relative to bloodstream forms.

### Interspecies comparison of developmental regulation

**Fig 2:**
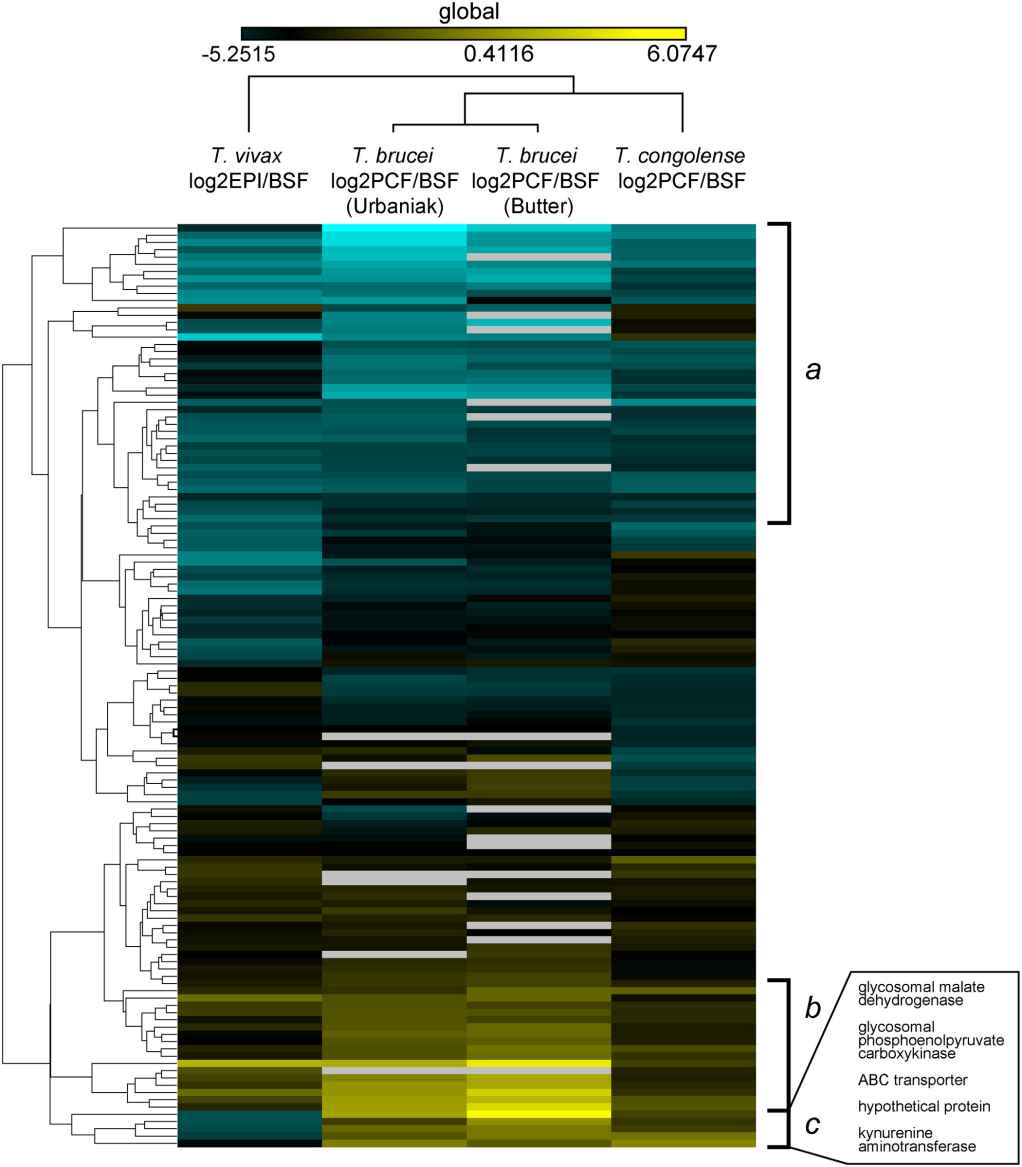
Fold changes in peptide abundance for 128 proteins when comparing insect stage (i.e. epimastigote (EPI) or procyclic form (PCF) and bloodstream forms (BSF), across three species. The phylogram shown at the top describes the overall similarity of the four datasets. The dendrogram at the left describes how the proteins cluster by expression profile. Blue shades indicate insect-stage expression; yellow shades indicate vertebrate-stage expression. Subset ‘*a’* are preferentially expressed in insect stages of all species. Subset ‘*b’* are enriched during the vertebrate stage of all species. Subset ‘*c’* is preferentially expressed in insect stages of *T*. *vivax* only.

Transcriptomic and proteomic data from diverse African trypanosomes indicate consistently that life stages differ with respect to primary energy metabolism. Previous studies of global gene expression *T*. *brucei* and *T*. *congolense* have shown developmental regulation of such genes, as well as those encoding the principal cell surface glycoproteins [12–13, 16–19, 21–22]. We would like to know how conserved such developmental regulation is through evolutionary time, and thus, how regulatory evolution might have contributed to phenotypic differences between species. Comparative genomics predicts that *T*. *vivax* lacks certain enigmatic components of *T*. *brucei* and *T*. *congolense* cell surfaces, such as the VSG-related transferrin receptor of bloodstream forms and procyclin, as well as some elements of Fam50 (see below) [26]. To relate global gene expression across the African trypanosomes, we compared our proteome with existing data sets for *T*. *brucei* [21–22] and *T*. *congolense* [13], calculating the fold change in abundance from the non-infective insect stage (i.e. epimastigote for *T*. *vivax* and procyclic form for *T*. *brucei* and *T*. *congolense*) to the bloodstream form, for each protein observed in all species (N = 128). Fig 2 compares relative peptide abundance across four proteomic datasets from three species.

Subset *a* contains proteins that are preferentially expressed in the bloodstream form in all species; the GO term for *glycolysis* (GO: 0006096; FDR = 6.4e^-06^) is enriched among these proteins. This indicates that the use of substrate-level phosphorylation as the dominant process for ATP generation in the bloodstream form is a consistent feature of African trypanosomes. Subset *b* contains proteins with the opposite expression profile, i.e. preferentially expressed in the non-infective insect stage in all species. Analysis of GO terms associated with these proteins shows that *proton-transporting ATP synthase activity* (GO:0046933; FDR = 1.8e^-03^), *ATP synthesis coupled proton transport* (GO:0015986; FDR = 1.8e^-03^) and *oxidation-reduction process* (GO:0055114; FDR = 3.1e^-03^) are enriched. This is consistent with the widespread use oxidative phosphorylation in the low-glucose environment of the insect vector to generate ATP via a proton-motive force across the mitochondrial membrane. Hence, at the broadest level, developmental regulation is conserved across all species, as their shared insect host will have predicted. However, there are obvious differences also.

**Fig 3:**
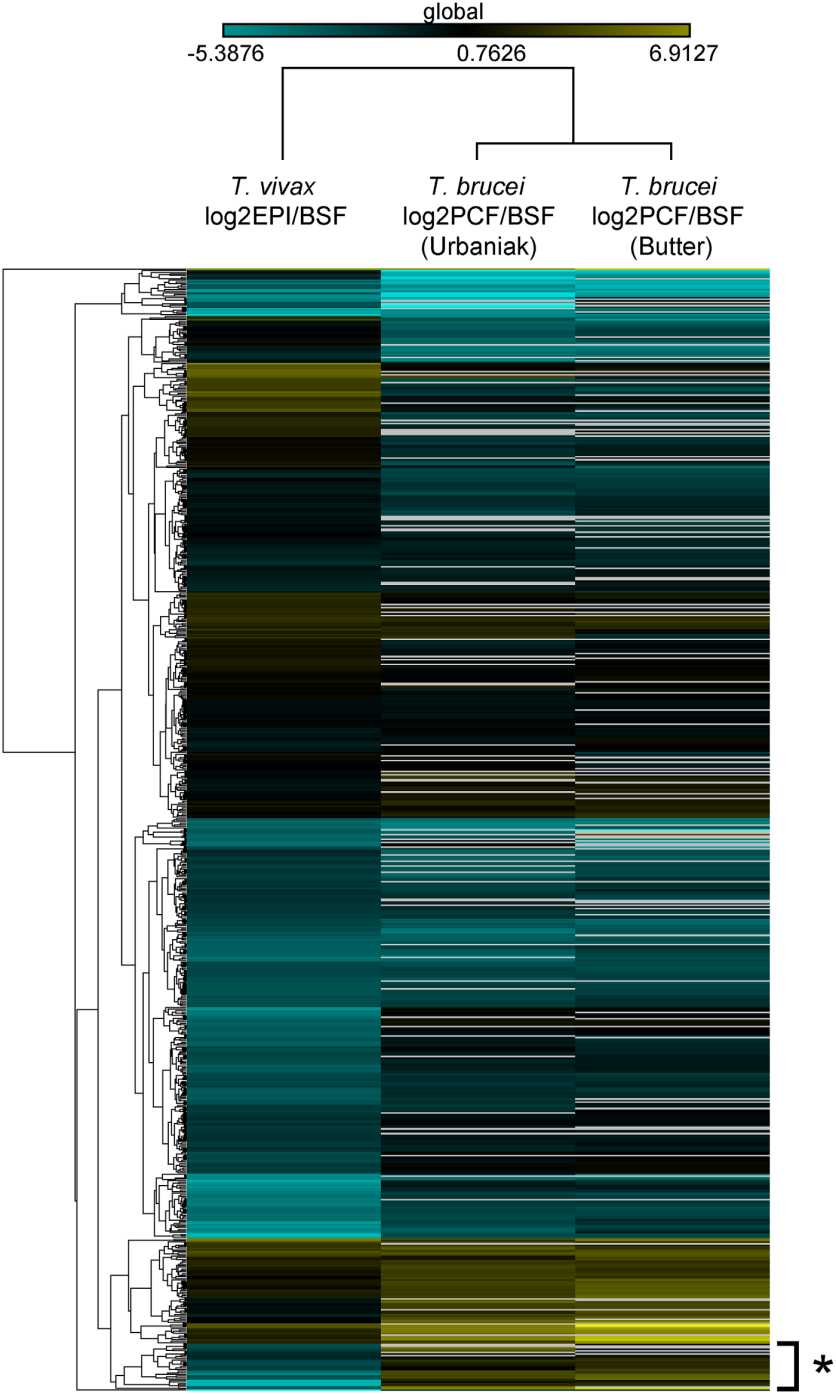
Fold changes in peptide abundance for 714 proteins when comparing insect stage (i.e. epimastigote (EPI) or procyclic form (PCF) and bloodstream forms (BSF), in *T*. *brucei* and *T*. *vivax*. The asterisk denotes an expanded cohort of insect-specific proteins in *T*. *vivax*, corresponding to subset *c* in Fig 2.

Against this background of conserved developmental regulation, we are interested in genes that are regulated differently in *T*. *vivax*, and which might contribute to its unique phenotypes. Subset *c* contains proteins that are significantly more abundant in the insect stages of *T*. *brucei* and *T*. *congolense* than the vertebrate stage, but preferentially expressed in *T*. *vivax* bloodstream forms. In the larger dataset of Fig 3, this cohort is expanded to 714 proteins by excluding *T*. *congolense* (for which proteome coverage is lowest); the number of proteins falling into subset *c* is increased to 27 and these are listed in Table 1. The expression profile of these proteins in *T*. *vivax* is not simply a lack of regulation or low expression generally, since many are highly abundant. Ten of the proteins in Table 1 appear in the top 10% of our proteome when ranked by abundance. Analysis of the GO terms associated with these proteins shows enrichment for *succinate-CoA ligase (GDP-forming) activity* (GO:0004776; FDR = 1.8e^-02^), *pyruvate dehydrogenase (acetyl-transferring) activity* (GO:0004739; FDR = 1.8e^-02^) and *glucose metabolic process* (GO:0006006; FDR = 2.8e^-02^). Hence, comparison of differentially expressed genes across African trypanosomes shows that much is conserved at the regulatory level but that important differences exist, even in the most essential physiology.

**Table 1:** Fold change in peptide abundance for 27 loci that show preferential expression in the bloodstream form of *T*. *vivax* and in the insect (procyclic) stage of *T*. *brucei*.

Gene ID	Product	*T*. *vivax*	*T*. *brucei* 1*	*T*. *brucei* 2**
TvY486_0902340	ABC transporter, putative	-2.44	2.66	2.85
TvY486_1007540	ABC transporter, putative	-1.20	2.75	3.11
TvY486_0904230	BRCT domain-containing hypothetical protein, conserved	-3.44	1.64	2.02
TvY486_0907550	CHAT domain-containing hypothetical protein, conserved	-0.68	3.15	0.81
TvY486_1007360	dihydrolipoamide acetyltransferase precursor, putative	-0.20	1.16	1.81
TvY486_0806870	dihydrolipoamide dehydrogenase, putative	-0.17	ND	1.37
TvY486_0604120	dynein heavy chain-containing protein, putative	-2.06	2.49	2.07
TvY486_1002790	eukaryotic translation initiation factor 5, putative	-3.66	1.56	1.06
TvY486_1014790	glycosomal malate dehydrogenase, putative	-1.28	4.09	6.07
TvY486_0039220	glycosomal phosphoenolpyruvate carboxykinase, putative	-1.31	1.84	3.63
TvY486_0101860	hypothetical protein, conserved	-0.57	2.63	ND
TvY486_0300460	hypothetical protein, conserved	-1.04	ND	1.31
TvY486_0303060	hypothetical protein, conserved	-0.68	2.06	2.02
TvY486_1011060	hypothetical protein, conserved	-1.74	2.67	2.15
TvY486_1111980	hypothetical protein, conserved	-0.96	1.51	1.80
TvY486_1114040	hypothetical protein, conserved (fragment)	-0.07	1.10	1.24
TvY486_0907650	kinesin, putative	-3.61	1.88	1.89
TvY486_1111670	kinesin-like protein, putative	-0.20	0.95	ND
TvY486_0901260	mitochondrial pyruvate carrier protein, putative	-0.74	1.02	2.43
TvY486_0702860	nitroreductase 4-like protein, putative	-0.93	2.32	3.11
TvY486_0100140	phosphoglycerate kinase, putative	-1.01	3.76	ND
TvY486_0604100	phosphoglycerate kinase, putative	-1.28	3.76	ND
TvY486_0301070	pyruvate dehydrogenase E1 beta subunit, putative	-0.77	1.00	1.70
TvY486_1012350	pyruvate dehydrogenase E1 component alpha subunit, putative	-0.65	0.95	1.89
TvY486_0902590	RNA binding protein, putative	-3.39	1.09	1.52
TvY486_1007240	succinyl-CoA ligase [GDP-forming] beta-chain, putative	-0.96	1.80	1.76
TvY486_0301600	succinyl-CoA synthetase alpha subunit, putative	-1.01	1.49	1.74

**Note:** Fold change in peptide abundance is calculated as log2(epimastigote/bloodstream form) for *T*. *vivax* and log2(procyclic/bloodstream form) for *T*. *brucei*.

* Urbaniak et al. 2012

** Butter et al. 2013.

ND: no data.

### Expression of *T*. *vivax*-specific, cell surface-expressed gene families

**Fig 4:**
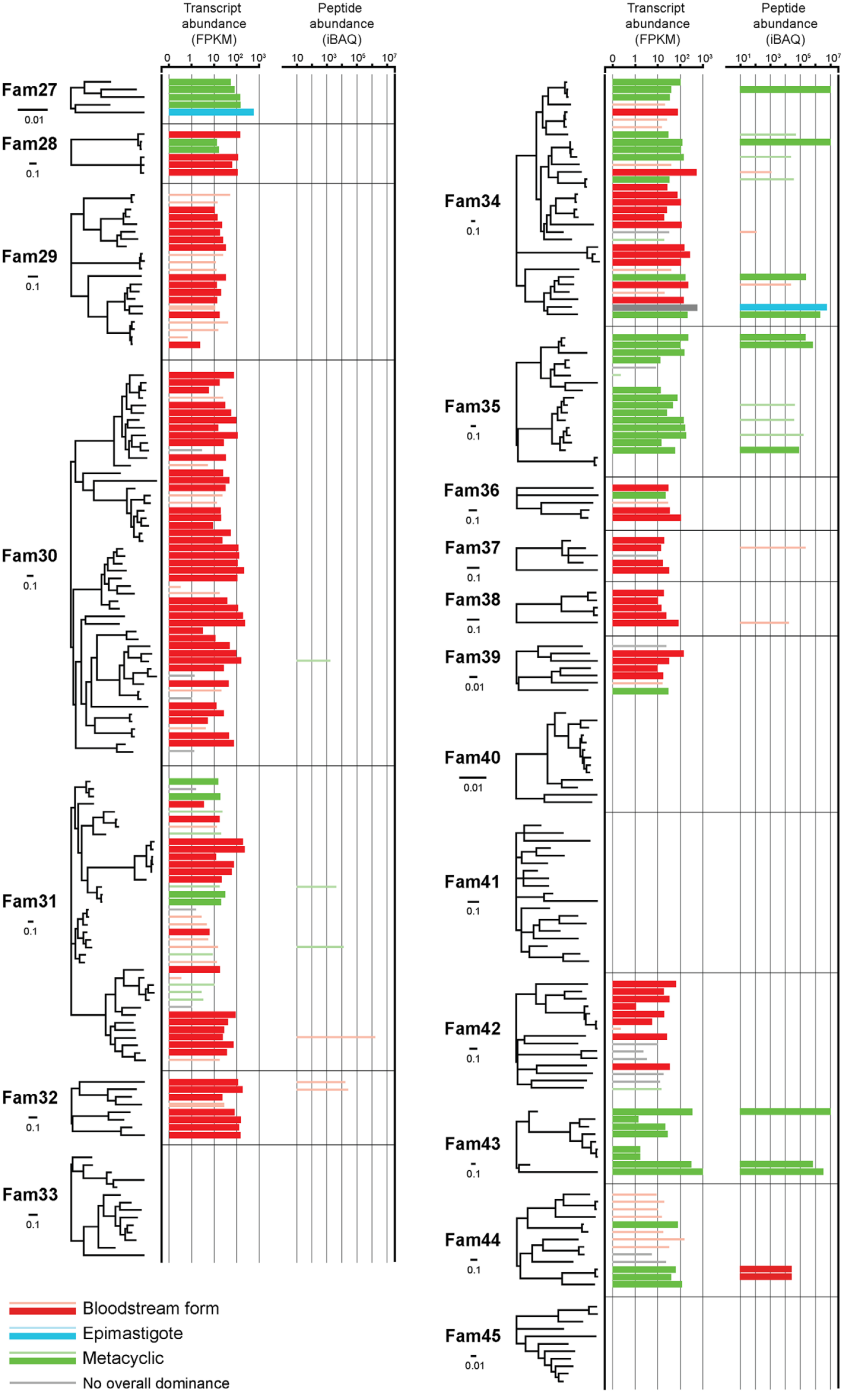
Differential expression of *Trypanosoma vivax*-specific genes belonging to Cell Surface Phylome families 27–45. The maximum likelihood phylogenies of 19 CSP families unique to *T*. *vivax* are shown at left. Node labels are omitted for clarity. Each terminal tip corresponds to a locus. Transcript abundance and peptide abundance are shown adjacent to each tip by horizontal bars color-coded by stage. Transcripts or peptides that also showed significant differential expression, (as defined in the text), are indicated by bold bars.

We have previously identified several gene families, known as Fam27-45, that are predicted to encode cell surface proteins and which, being unique to *T*. *vivax*, might distinguish the parasite from *T*. *brucei* and *T*. *congolense* [26]. Fam27-45 are among the most highly expressed genes and these data are extracted in Table 2. Many of the CSP families unique to *T*. *vivax* also appear to be developmentally regulated at the transcript level. For example, Fam27 (five paralogs), Fam35 (11 paralogs) and Fam 43 (five paralogs) are preferentially transcribed in insect stages. Conversely, Fam29 (13 paralogs), Fam30 (38 paralogs) and Fam32 (seven paralogs) are preferentially transcribed in bloodstream forms. Indeed, rarely are transcripts belonging to one of the families found throughout the lifecycle; Fam34 (25 paralogs) being one such case. Fig 4 summarizes the evidence for differential expression of Fam27-45 at the transcript and peptide level. In four cases, (Fams 33, 40, 41 and 45), both transcriptomic or proteomic evidence for expression is lacking and we conclude that these sequence families do not encode protein-coding genes (and so should be removed from the CSP). As expected, proteomic evidence for gene expression is not as prevalent as transcriptomic data, although it generally corroborates the latter when it is available.

**Table 2:** Preferential expression of *T*. *vivax*-specific Cell Surface Phylome (CSP) gene families based on fold change (FC) in transcript (a) and peptide (b) abundance across three life cycle stages.

**a)**	**Gene ID**	**CSP ID**	**Value 1**	**Stage**	**Value 2**	**Stage**	**FC**	**Max. stage**
	TvY486_0019430	Fam27	97.6	BSF	2488.1	EPI	21.83	EPI
	TvY486_0038280	Fam27	74.4	BSF	325.6	MET	4.54	MET
	TvY486_0038300	Fam27	137.6	BSF	606.6	MET	4.58	MET
	TvY486_0043530	Fam27	71.0	BSF	317.2	MET	4.66	MET
	TvY486_0044810	Fam27	126.3	BSF	701.7	MET	6.12	MET
	TvY486_0001150	Fam28	43.3	MET	10.5	EPI	4.19	MET
	TvY486_0005050	Fam28	310.0	BSF	17.7	EPI	17.09	BSF
	TvY486_0017990	Fam28	274.6	BSF	12.5	EPI	19.89	BSF
	TvY486_0019710	Fam28	60.5	MET	14.2	EPI	4.38	MET
	TvY486_0030920	Fam28	203.3	BSF	4.7	MET	29.40	BSF
	TvY486_0040160	Fam28	488.7	BSF	28.5	EPI	16.82	BSF
	TvY486_0010970	Fam29	3.4	BSF	0.7	EPI	4.88	BSF
	TvY486_0011750	Fam29	72.4	BSF	13.5	MET	5.88	BSF
	TvY486_0013730	Fam29	65.3	BSF	17.0	MET	3.76	BSF
	TvY486_0014410	Fam29	37.6	BSF	9.2	EPI	4.12	BSF
	TvY486_0014700	Fam29	85.2	BSF	23.3	EPI	3.50	BSF
	TvY486_0015070	Fam29	122.2	BSF	41.5	MET	2.42	BSF
	TvY486_0015280	Fam29	70.0	BSF	16.5	MET	4.33	BSF
	TvY486_0030720	Fam29	126.5	BSF	26.5	MET	5.09	BSF
	TvY486_0033210	Fam29	33.0	BSF	9.7	MET	3.10	BSF
	TvY486_0036530	Fam29	42.2	BSF	11.4	MET	3.57	BSF
	TvY486_0040000	Fam29	124.6	BSF	29.8	MET	4.26	BSF
	TvY486_0040440	Fam29	45.6	BSF	15.1	MET	2.53	BSF
	TvY486_0000090	Fam30	63.0	BSF	8.8	EPI	8.08	BSF
	TvY486_0001730	Fam30	62.8	BSF	13.1	EPI	5.13	BSF
	TvY486_0001740	Fam30	191.8	BSF	33.7	EPI	6.29	BSF
	TvY486_0003600	Fam30	301.9	BSF	9.1	EPI	25.58	BSF
	TvY486_0003670	Fam30	233.4	BSF	5.5	EPI	29.35	BSF
	TvY486_0004540	Fam30	25.7	BSF	8.2	EPI	2.71	BSF
	TvY486_0005120	Fam30	20.6	BSF	5.1	EPI	4.07	BSF
	TvY486_0005240	Fam30	165.2	BSF	7.5	EPI	19.95	BSF
	TvY486_0005910	Fam30	266.6	BSF	6.6	EPI	28.49	BSF
	TvY486_0006060	Fam30	127.5	BSF	11.4	EPI	12.14	BSF
	TvY486_0006100	Fam30	101.4	BSF	14.9	EPI	7.67	BSF
	TvY486_0007350	Fam30	33.6	BSF	11.0	MET	2.58	BSF
	TvY486_0007870	Fam30	354.5	BSF	10.2	EPI	26.25	BSF
	TvY486_0008460	Fam30	156.8	BSF	11.3	MET	14.43	BSF
	TvY486_0011830	Fam30	129.1	BSF	30.0	EPI	4.44	BSF
	TvY486_0014950	Fam30	372.6	BSF	34.0	EPI	11.94	BSF
	TvY486_0016140	Fam30	531.7	BSF	40.6	MET	13.77	BSF
	TvY486_0016150	Fam30	474.4	BSF	43.0	EPI	12.00	BSF
	TvY486_0020240	Fam30	682.5	BSF	29.7	EPI	20.43	BSF
	TvY486_0020730	Fam30	95.4	BSF	9.1	EPI	11.48	BSF
	TvY486_0021030	Fam30	162.7	BSF	14.1	EPI	12.45	BSF
	TvY486_0022710	Fam30	195.4	BSF	18.6	EPI	11.54	BSF
	TvY486_0023840	Fam30	42.9	BSF	11.5	MET	3.60	BSF
	TvY486_0027870	Fam30	107.7	BSF	21.5	EPI	5.39	BSF
	TvY486_0029560	Fam30	248.4	BSF	23.5	EPI	11.56	BSF
	TvY486_0029680	Fam30	36.8	BSF	3.6	EPI	11.32	BSF
	TvY486_0029750	Fam30	156.6	BSF	21.5	EPI	8.19	BSF
	TvY486_0031200	Fam30	22.8	BSF	5.6	EPI	4.13	BSF
	TvY486_0031450	Fam30	211.0	BSF	51.4	EPI	4.15	BSF
	TvY486_0032170	Fam30	169.2	BSF	19.3	EPI	9.80	BSF
	TvY486_0032760	Fam30	234.1	BSF	44.5	EPI	5.74	BSF
	TvY486_0033670	Fam30	200.1	BSF	16.0	EPI	13.27	BSF
	TvY486_0036500	Fam30	71.8	BSF	9.2	EPI	8.83	BSF
	TvY486_0037590	Fam30	347.9	BSF	19.3	EPI	17.39	BSF
	TvY486_0037600	Fam30	120.6	BSF	18.9	EPI	7.16	BSF
	TvY486_0038800	Fam30	254.9	BSF	14.6	EPI	16.98	BSF
	TvY486_0041770	Fam30	71.6	BSF	11.9	EPI	6.72	BSF
	TvY486_0043800	Fam30	167.5	BSF	7.0	EPI	21.02	BSF
	TvY486_0044700	Fam30	91.2	BSF	11.0	EPI	9.32	BSF
	TvY486_0000210	Fam31	510.7	BSF	16.8	MET	24.29	BSF
	TvY486_0007400	Fam31	186.1	BSF	15.1	EPI	13.11	BSF
	TvY486_0008000	Fam31	168.5	BSF	9.2	EPI	17.61	BSF
	TvY486_0010510	Fam31	62.2	BSF	23.2	EPI	2.03	BSF
	TvY486_0011600	Fam31	80.1	MET	30.1	EPI	1.99	MET
	TvY486_0014260	Fam31	202.0	BSF	24.6	MET	9.22	BSF
	TvY486_0017860	Fam31	148.6	MET	34.8	EPI	4.39	MET
	TvY486_0020480	Fam31	174.0	BSF	9.8	EPI	17.24	BSF
	TvY486_0023090	Fam31	949.0	BSF	34.7	MET	22.78	BSF
	TvY486_0023590	Fam31	227.5	BSF	24.3	MET	10.42	BSF
	TvY486_0026820	Fam31	25.4	BSF	8.7	EPI	2.40	BSF
	TvY486_0027490	Fam31	168.7	BSF	12.0	EPI	14.58	BSF
	TvY486_0029930	Fam31	54.4	MET	14.5	EPI	3.64	MET
	TvY486_0034320	Fam31	27.2	BSF	8.9	MET	2.59	BSF
	TvY486_0038020	Fam31	72.6	MET	18.4	EPI	3.91	MET
	TvY486_0040710	Fam31	94.1	BSF	27.6	EPI	3.13	BSF
	TvY486_0042250	Fam31	102.9	BSF	31.7	EPI	2.89	BSF
	TvY486_0042880	Fam31	118.3	BSF	9.6	EPI	13.10	BSF
	TvY486_0044520	Fam31	114.5	MET	31.7	EPI	3.44	MET
	TvY486_0045910	Fam31	120.3	BSF	10.3	EPI	12.56	BSF
	TvY486_0002120	Fam32	377.9	BSF	40.8	EPI	10.31	BSF
	TvY486_0004160	Fam32	503.7	BSF	53.8	EPI	10.41	BSF
	TvY486_0010310	Fam32	155.8	BSF	29.3	EPI	5.82	BSF
	TvY486_0025570	Fam32	193.1	BSF	5.3	MET	26.77	BSF
	TvY486_0027130	Fam32	285.5	BSF	32.7	EPI	9.78	BSF
	TvY486_0042890	Fam32	225.2	BSF	69.7	EPI	2.86	BSF
	TvY486_0043780	Fam32	421.4	BSF	43.9	EPI	10.64	BSF
	TvY486_0001120	Fam34	24.7	BSF	154.1	MET	6.96	MET
	TvY486_0004310	Fam34	177.4	BSF	61.9	EPI	2.31	BSF
	TvY486_0004790	Fam34	421.5	BSF	2256.7	MET	5.86	MET/EPI
	TvY486_0007010	Fam34	380.0	BSF	90.4	EPI	4.29	BSF
	TvY486_0008750	Fam34	84.4	BSF	25.6	EPI	2.95	BSF
	TvY486_0009950	Fam34	981.2	BSF	27.7	EPI	26.48	BSF
	TvY486_0013520	Fam34	46.6	BSF	233.9	MET	5.42	MET
	TvY486_0017330	Fam34	15.9	BSF	380.9	MET	20.97	MET
	TvY486_0018370	Fam34	317.9	BSF	81.8	MET	3.83	BSF
	TvY486_0019090	Fam34	9.1	BSF	261.1	MET	23.52	MET
	TvY486_0020950	Fam34	244.7	BSF	26.4	MET	10.34	BSF
	TvY486_0021420	Fam34	403.2	BSF	145.4	EPI	2.17	BSF
	TvY486_0022140	Fam34	23.7	BSF	185.0	MET	8.77	MET
	TvY486_0024340	Fam34	2178.6	BSF	96.6	EPI	20.21	BSF
	TvY486_0029710	Fam34	41.0	BSF	174.2	MET	4.35	MET
	TvY486_0029720	Fam34	50.6	BSF	667.5	MET	13.84	MET
	TvY486_0030370	Fam34	333.3	BSF	73.7	EPI	4.74	BSF
	TvY486_0032770	Fam34	52.9	BSF	617.6	MET	12.56	MET
	TvY486_0034300	Fam34	26.2	BSF	143.1	MET	6.00	MET
	TvY486_0035000	Fam34	131.5	BSF	41.0	EPI	2.82	BSF
	TvY486_0035610	Fam34	227.1	BSF	81.5	MET	2.18	BSF
	TvY486_0035970	Fam34	285.4	BSF	31.5	EPI	10.10	BSF
	TvY486_0039910	Fam34	465.4	BSF	2354.6	MET	5.47	MET/EPI
	TvY486_0044510	Fam34	21.1	BSF	392.5	MET	17.81	MET
	TvY486_0900430	Fam34	859.1	BSF	44.0	EPI	18.37	BSF
	TvY486_0004880	Fam35	0.3	BSF	31.0	MET	43.79	MET
	TvY486_0006390	Fam35	1.0	BSF	151.1	MET	51.87	MET
	TvY486_0010560	Fam35	3.9	BSF	539.8	MET	50.36	MET
	TvY486_0011320	Fam35	58.0	MET	8.0	EPI	8.12	MET
	TvY486_0011480	Fam35	1.5	BSF	451.0	MET	67.73	MET
	TvY486_0014750	Fam35	0.7	BSF	564.1	MET	92.02	MET
	TvY486_0016780	Fam35	4.1	BSF	50.9	MET	13.22	MET
	TvY486_0030340	Fam35	1.2	BSF	206.6	MET	55.65	MET
	TvY486_0030570	Fam35	1.6	BSF	394.5	MET	63.36	MET
	TvY486_0039920	Fam35	178.2	MET	25.3	EPI	7.93	MET
	TvY486_0041300	Fam35	2.1	BSF	769.3	MET	72.56	MET
	TvY486_0044010	Fam35	4.1	BSF	669.1	MET	53.79	MET
	TvY486_0504020	Fam35	0.8	BSF	319.7	MET	75.03	MET
	TvY486_0004900	Fam36	122.8	BSF	27.1	EPI	4.76	BSF
	TvY486_0019680	Fam36	245.7	BSF	26.9	EPI	10.19	BSF
	TvY486_0034550	Fam36	48.4	BSF	139.7	MET	2.34	MET
	TvY486_0039550	Fam36	136.2	BSF	21.4	EPI	7.11	BSF
	TvY486_0008720	Fam37	63.7	BSF	16.8	EPI	3.68	BSF
	TvY486_0010370	Fam37	112.0	BSF	31.3	MET	3.38	BSF
	TvY486_0037640	Fam37	107.2	BSF	37.4	MET	2.31	BSF
	TvY486_0044820	Fam37	86.5	BSF	15.0	MET	6.38	BSF
	TvY486_0012580	Fam38	40.9	BSF	13.8	EPI	2.47	BSF
	TvY486_0018310	Fam38	79.2	BSF	13.0	MET	6.79	BSF
	TvY486_0019140	Fam38	106.4	BSF	19.3	MET	6.05	BSF
	TvY486_0038940	Fam38	96.2	BSF	31.7	MET	2.56	BSF
	TvY486_0045330	Fam38	234.9	BSF	36.6	MET	7.18	BSF
	TvY486_0014250	Fam39	364.1	BSF	82.2	EPI	4.61	BSF
	TvY486_0036690	Fam39	136.8	BSF	35.2	MET	3.84	BSF
	TvY486_0041320	Fam39	22.0	BSF	6.7	EPI	2.95	BSF
	TvY486_0044730	Fam39	59.8	BSF	19.5	EPI	2.61	BSF
	TvY486_1101450	Fam39	53.8	BSF	183.7	MET	3.14	MET
	TvY486_0012130	Fam42	85.4	BSF	28.7	EPI	2.48	BSF
	TvY486_0012790	Fam42	90.8	BSF	10.1	EPI	10.03	BSF
	TvY486_0017880	Fam42	130.3	BSF	26.3	EPI	5.32	BSF
	TvY486_0019770	Fam42	17.8	BSF	1.8	EPI	10.99	BSF
	TvY486_0027860	Fam42	2.0	BSF	0.2	EPI	11.25	BSF
	TvY486_0028850	Fam42	146.1	BSF	16.4	MET	9.96	BSF
	TvY486_0039500	Fam42	194.4	BSF	37.7	EPI	5.60	BSF
	TvY486_1109320	Fam42	109.0	BSF	30.5	EPI	3.38	BSF
	TvY486_0002350	Fam43	0.2	BSF	5.1	MET	19.71	MET
	TvY486_0017520	Fam43	6.4	MET	0.8	EPI	8.51	MET
	TvY486_0017890	Fam43	2.7	BSF	1245.9	MET	78.16	MET
	TvY486_0021360	Fam43	0.5	BSF	129.6	MET	64.82	MET
	TvY486_0027480	Fam43	9.6	BSF	5992.2	MET	86.31	MET
	TvY486_0031050	Fam43	4.2	MET	0.5	EPI	9.41	MET
	TvY486_0040930	Fam43	57.1	MET	8.3	EPI	7.79	MET
	TvY486_0044590	Fam43	5.5	BSF	1708.8	MET	68.43	MET
	TvY486_0005330	Fam44	116.6	BSF	505.9	MET	4.48	MET
	TvY486_0012520	Fam44	62.3	BSF	266.6	MET	4.40	MET
	TvY486_0027940	Fam44	59.5	BSF	277.7	MET	4.94	MET
	TvY486_0042450	Fam44	40.3	BSF	162.7	MET	4.06	MET
**(b)**	**Gene ID**	**CSP ID**	**Peptide**	**iBAQ:**			**FC**	**Max. stage**
			**count**	BSF	EPI	MET		
	TvY486_0013520	Fam34	5	3598.7	1887.8	20547.2	10.88	MET
	TvY486_0032770	Fam34	19	2030.8	817.7	7244.1	8.86	MET
	TvY486_0029720	Fam34	3	15149	10247.4	90551.7	8.84	MET
	TvY486_0039910	Fam34	5	76451	293863.7	95768.8	3.84	MET
	TvY486_0036040	Fam34	11	91.91	103.0	966.1	10.51	MET
	TvY486_0034300	Fam34	17	534.44	840.1	1175.6	2.2	MET
	TvY486_0041300	Fam35	4	5943	4140.9	27129.1	6.55	MET
	TvY486_0039920	Fam35	3	275.32	783.5	6109.4	22.19	MET
	TvY486_0017890	Fam43	4	1711.5	2652.5	52918.0	30.92	MET
	TvY486_0027480	Fam43	6	16573	11934.9	237265.1	19.88	MET
	TvY486_0044590	Fam43	5	41982	58347.1	1211175	28.85	MET
	TvY486_0012520	Fam44	8	2370.2	2182.6	165.9	14.28	BSF

NB: For transcripts, significant differential expression is estimated from pairwise comparisons of life cycle stages using Cufflinks. For peptides, iBAQ abundance is compared across all three stages simultaneously. q < 0.05 in all cases. Developmental stages are abbreviated with BSF (bloodstream form), EPI (epimastigote), MET (metacyclic).

The best supported cases for developmental regulation concern the metacyclic-specific expression of Fam34, 35 and 43. We observed 34 distinct Fam34 transcripts and 24 of these were differentially expressed; 12 in the metacyclic and another 12 in the bloodstream form. However, the proteomic evidence indicates more selective developmental regulation; of 11 Fam34 proteins that were observed, five were preferentially expressed and all in the metacyclic stage (FC between 2.2–10.8).

Of 17 distinct Fam35 transcripts, 11 were differentially expressed; all were significantly more abundant in the metacyclic stage (FC between 13.2–92.0). Proteomic data support this view; of the six Fam35 peptides observed, all were most abundant in the metacyclic stage and two significantly so (TvY486_0041300 (FC = 6.55) and TvY486_0039920 (FC = 22.19)).

We observed seven distinct Fam43 transcripts and of these five were differentially expressed, all most abundant in the metacyclic (FC between 19.7–86.3). All three Fam43 peptides that were observed were preferentially expressed in the metacyclic stage (FC between 19.8–30.9). Hence, these results indicate that the putative *T*. *vivax*-specific gene families are (mostly) genuine protein-coding sequences, and are often developmentally regulated at the transcript and (where observed) protein level.

### Expression of ‘Fam50’ genes

Fam50 is a CSP gene family that includes the BARP genes of *T*. *brucei* and the GARP and CESP genes of *T*. *congolense*, known to be preferentially expressed on their respective cell surfaces during the insect stages [25, 38–40], as well as various, currently uncharacterized, genes that may also be transcribed preferentially during insect stages [41]. The genomic complement of Fam50 genes in *T*. *vivax* is smaller and less diverse than those of the other species, which may reflect the simpler existence of *T*. *vivax* in the tsetse fly [26]. Our transcriptomic data include all 17 *T*. *vivax* Fam50 genes, 13 of which are transcribed most abundantly in the insect stages (see S1 Table). Six transcripts are significantly more abundant in the epimastigote or metacyclic stage relative to bloodstream forms (Table 3a) and one of these was confirmed by the proteomic data (i.e. TvY486_0012620). In total, five proteins were detected and all were differentially expressed (Table 3b); four were most abundant in the insect (FC between 2.2 and 4.2). A single protein (corresponding to TvY486_0001140) was significantly more abundant in the bloodstream form. Thus, while transcriptomic data seems to be a poor predictor of differential expression of Fam50 proteins, perhaps suggesting the highly dynamic promotion and repression of Fam50 variants, there is good evidence for developmental regulation of both Fam50 transcripts and peptides, largely in preference for the insect stages and so consistent with observations in other species.

**Table 3:** Preferential expression of (a) transcripts and (b) peptides belonging to Fam50 (BARP-like genes) in specific life cycle stages.

**a)**	**Gene ID**	**FPKM:**			**FC**	**Max. stage**
		BSF	EPI	MET		
	TvY486_0000390	383.5	2159.8	1826.9	6.22	EPI
	TvY486_0012620	481.9	2456.5	2497.4	5.52	EPI/MET
	TvY486_0044170	381.3	2190.3	1869.5	6.36	EPI
	TvY486_1000710	299.1	1777.0	1465.8	6.61	EPI
	TvY486_1001160	439.7	1936.2	2077.4	4.57	EPI/MET
	TvY486_1114940	347.6	2018.1	2138.0	6.44	EPI/MET
**b)**	**Gene ID**	**iBAQ:**			**FC**	**Max. stage**
		BSF	EPI	MET		
	TvY486_0001140	**693301.4**	163534.9	100466.4	6.90	BSF
	TvY486_0012620	135508.0	**265088.1**	**292408.3**	2.16	EPI/MET
	TvY486_0016400	71617.0	**159017.0**	98083.8	2.22	EPI
	TvY486_0016530*	179679.6	**584272.8**	139906.5	4.18	EPI
	TvY486_1114940	11074.5	**40556.9**	17551.1	3.66	EPI

NB: Developmental stages are abbreviated with BSF (bloodstream form), EPI (epimastigote), MET (metacyclic). Differential expression is described by fold change (FC) in epimastigotes relative to bloodstream forms. q < 0.05 in all cases. * Note that these two coding sequences are near-identical and so cross-mapping of reads could affect their FPKM values. * Note that peptides mapping to this gene could equally map to two other gene models (TvY486_1001150/TvY486_1001160) that are identical at this position.

### Expression of *VSG*-like genes

The bloodstream forms of African trypanosomes are defined partly by the expression of a VSG coat on the cell surface. In our analysis, we observed 89 distinct VSG transcripts with q < 0.05 (S1 Table), of which 61 were most abundant in bloodstream forms; however, most of these were observed at very low levels. There were 12 transcripts displaying significant preferential expression in the bloodstream stage (FC between 2.5–110.9; Table 4a). In our proteomic analysis we recorded nine distinct VSG sequences, of which three are represented by a single peptide and so unquantified (S2 Table). Of the remaining six (Table 4b), three were most abundant in bloodstream forms, including the two putative active VSG and a third sequence (TvY486_0000810, or identical paralog) that was expressed at a much lower level but still preferentially in bloodstream forms (FC = 5.7). A fourth VSG was expressed preferentially in the metacyclic stage (TvY486_0027560; FC = 2.8). Finally, two VSG were most abundant in the epimastigote; one of these (TvY486_0001860; FC = 4.3) was differentially expressed and the second nearly so (TvY486_0041140; FC = 2.1; q = 0.066). Notably, these low abundance VSG expressed in epimastigotes belong to a *T*. *vivax*-specific VSG-like family (Fam25), which is not seen in other African trypanosomes.

**Table 4:** Preferential expression of (a) transcripts and (b) peptides belonging to variant surface glycoprotein-like genes.

**a)**	**Gene ID**	**CSP ID**		**BSF**	**EPI**	**MET**	**FC**	**Max. stage**
	TvY486_0009580	Fam24		34180.1	245.0	301	109.2	BSF
	TvY486_0018880	Fam24		26001.7	20.1	20.8	106.7	BSF
	TvY486_0002880	Fam23		22.7	2.5	3.20	10.0	BSF
	TvY486_0027380	Fam23		201.1	30.1	37.3	7.5	BSF
	TvY486_0028170	Fam23		212.4	32.3	40.0	7.3	BSF
	TvY486_0025310	Fam23		188.6	36.4	-	5.6	BSF
	TvY486_0019720	Fam23		107.2	23.9	38.0	4.6	BSF
	TvY486_0006330	Fam23		147.1	33.9	42.1	4.4	BSF
	TvY486_0039540	Fam23		848.1	203.8	-	4.2	BSF
	TvY486_0010070	Fam23		69.3	18.2	21.2	3.7	BSF
	TvY486_0011440	Fam23		54.0	16.6	16.2	2.9	BSF
	TvY486_0043550	Fam23		97.3	30.1	-	2.8	BSF
	TvY486_0040490	Fam23		111.9	36.9	36.7	2.5	BSF
	**Gene ID**	**CSP ID**	**Peptide**	**iBAQ:**			**FC**	**Max. stage**
**b)**			**count**	BSF	EPI	MET		
	TvY486_0018880	Fam24	15	22283050	817644.1	454910.3	48.9	BSF
	TvY486_0009580	Fam24	22	217715149	2295147	1824989	119.3	BSF
	TvY486_0000810*	Fam23	2	51810.2	21467.7	9041.9	5.7	BSF
	TvY486_0001860	Fam25	1	44940.7	113671.8	26300.0	4.3	EPI
	TvY486_0027560#	Fam24	1	44757.3	54295.2	124747.7	2.7	MET
	TvY486_0041140	Fam25	1	49076.9	117452	61305.4	2.3	EPI

NB: For transcript abundance, details are provided for pairwise comparisons that resulted in significant differences (in some cases involving a single gene in two comparisons). Developmental stages are abbreviated with BSF (bloodstream form), EPI (epimastigote), MET (metacyclic). Differential expression is described by fold change (FC). q < 0.05 in all cases. Peptide count refers to the total number of unique peptides observed for a given protein. Note that peptides mapping to * could equally map to other, identical gene models: TvY486_0000330; TvY486_0028900; TvY486_0002800; TvY486_0003200; TvY486_0005340; TvY486_0005830; TvY486_0008280; TvY486_0031250; TvY486_0031440; TvY486_0039630; TvY486_0042400. Also peptides mapping to # could equally map to these other, identical gene models: TvY486_0009200; TvY486_0015630; TvY486_0016270; TvY486_0031530; TvY486_0031920; TvY486_0031980; TvY486_0042360

## Discussion

We have produced transcriptomes and proteomes for three different developmental forms of *T*. *vivax*, and identified the transcripts and peptides that are significantly enriched in each. These data provide the first profile of global gene expression and developmental regulation throughout the complete *T*. *vivax* life cycle. The profile suggests a situation broadly similar to that already observed in *T*. *brucei* and *T*. *congolense*, though with significant distinctions, not least further evidence for developmental regulation of species-specific cell surface glycoproteins in both the vertebrate and insect stages of *T*. *vivax*.

Such are their sensitivities, transcriptomic methods typically provide much greater coverage of the genome than do proteomic methods. This might be more pronounced for trypanosomatids since they constitutively express all genes within polycistronic transcripts and regulate protein expression post-transcriptionally [27]. Thus, 85.1% of *T*. *vivax* genes are represented in our transcriptome, but few transcripts are unique to a particular life stage and the proportion of differentially expressed transcripts is only 11.2%. By contrast, the proteome represents only 16.3% of all genes, but in 798 cases where differential expression could be assessed, 74.6% of peptides show significant differential expression and these are unique to one life stage. It may be that developmentally regulated proteins are also particularly abundant, certainly this is true for the components of stage-specific cell surface coats, and this would cause differentially expressed peptides to be overrepresented within the proteome. Previous proteomic studies for *T*. *brucei* have reported more proteins than we have found here for *T*. *vivax* (i.e. 3553 [21] and 3458 [22]) but a smaller proportion with significant differential expression, i.e. 24.8%/39.2% respectively. A proteomic analysis of *T*. *congolense* identified 1291 proteins, of which 21.5% displayed significant differential expression [13]. Hence, it may be that greater sensitivity would reduce the proportion of cases showing differential expression if low abundance proteins are more likely to be constitutively expressed.

Previously, comparison of the *T*. *vivax* genome sequence with those of *T*. *brucei* and *T*. *congolense* demonstrated that there are more than 2000 genes that are only present in *T*. *vivax* [11]. Their specificity, and typically the absence of any recognizable protein domains, make these genes obscure. Nonetheless, they appear to be genuine, since we found a lack of protein-coding evidence in only a few cases. One defining feature is that they comprise multi-copy gene families (Fam27-45 of the CSP) that are thought to be expressed on the cell surface based on the presence of a putative signal peptide, transmembrane domain and/or glycerophosphosinositol anchor in their predicted protein sequence [26]. Comparative genomics also showed that *T*. *vivax* lacked procyclin, the canonical cell surface glycoprotein of *T*. *brucei* and *T*. *congolense* insect stages [42]. This is consistent with *T*. *vivax* lacking a procyclic stage in the insect mid-gut and raises the question of what coats the *T*. *vivax* surface if not procyclin. Clearly, the abundant *T*. *vivax-*specific gene families offer plausible candidates for the role, but it could also be filled by Fam50; which has been shown to include various surface glycoproteins expressed during the insect stages of *T*. *brucei* and *T*. *congolense* [26].

Certainly, our data indicate that multiple Fam50 proteins are expressed in *T*. *vivax* and preferentially expressed in the epimastigote, while several transcripts (belonging to different loci) were significantly enriched in both epimastigotes and metacyclics. The fact that the transcripts and peptides are not derived from the same loci may suggest that different Fam50 genes became activated in the period between our preparation of RNA and protein. This is not the situation we observe for VSG, for which the identity of enriched transcripts and peptides largely match, suggesting that regulation of Fam50 gene expression is highly dynamic with multiple isoforms being promoted and repressed over short intervals. The presence of transcripts in the metacyclic stage at levels comparable to the epimastigote levels may be an artefact (i.e. residual epimastigotes in the metacyclic preparation), since Fam50 peptides in metacyclics are sparse and comparable in abundance to bloodstream forms. In short, the expression of multiple Fam50 proteins in the *T*. *vivax* epimastigote supports the view derived from *T*. *brucei* and *T*. *congolense* that this is a conserved family of glycoproteins performing diverse roles in the insect stages of the life cycle. Given that BARP in *T*. *brucei* and CESP in *T*. *congolense* are cell-surface glycoproteins [25, 40], Fam50 homologs are therefore probably a prominent component of the epimastigote cell surface in *T*. *vivax*.

Although Fam50 is preferentially expressed in epimastigotes, other multi-copy families, including the various *T*. *vivax*-specific cases, seldom are. This study has confirmed that most *T*. *vivax*-specific gene families are expressed. In the cases of Fam33, 40, 41 and 45, which should now be discounted from the CSP, the apparent lack of transcription raises the question of what function these repetitive non-coding sequences might perform. Three *T*. *vivax*-specific gene families (Fam34, 35 and 43) are very strongly enriched in the metacyclic stage, which is intriguing because in *T*. *brucei* and *T*. *congolense* the metacyclic coat is characterized by VSG. While metacyclic VSG are replaced by other VSG upon differentiation into bloodstream forms, and so are temporally distinct, there is no metacyclic-specific cohort of VSG sequences [8, 11]. VSG may also be present on *T*. *vivax* metacyclics, since we observed a low abundance VSG protein preferentially expressed in metacyclics (TvY486_0027560). However, assuming that Fam34, 35 and 43 are expressed on the cell surface as predicted, it is clear that the infective form of *T*. *vivax* has a qualitatively different surface architecture to the other species, with a considerable non-VSG component.

The same could be claimed for bloodstream forms also. Three families show exclusive enrichment in bloodstream forms at the transcript level (Fam28-30), though without proteomic support. This could indicate that our analysis lacked the sensitivity to detect them, perhaps because in bloodstream forms the superabundant VSG dominates the sequencing effort, making the detection of lower abundance proteins less effective than for either metacyclic or epimastigote. The presence of numerous non-VSG surface proteins might account for the observation that the *T*. *vivax* VSG coat is less dense than that of *T*. *brucei* [43], and VSG comprise a smaller proportion of cell surface-expressed *T*. *vivax* transcripts [14]. Assuming that their surface role is correct and they are confirmed as having preferential expression in bloodstream forms, these families are particularly interesting they have properties as surface antigens that could be targeted for vaccine development. Immune responses to the VSG do not provide lasting and comprehensive protection because of antigenic variation and the considerable structural diversity of the VSG repertoire. By contrast, Fam28-30 number not more than 30 paralogs, are less structurally diverse, and multiple transcripts are expressed at relatively high abundance, indicating that these families are not subject to antigenic variation, if monoallelic expression is a diagnostic feature of that process.

The VSG genes themselves present an expression profile typical of other African trypanosomes. VSG expression is regulated to produce a succession of structural variants that can evade specific immune responses but also prevent exposure of the total VSG structural repertoire to the host immune system, which would lead to a comprehensive immune response. Thus, VSG genes are expressed in a monoallelic fashion from the highly regulated context of a dedicated VSG expression site [44]. In their analysis of expressed sequence tags (EST) from different *T*. *congolense* life stages, Helm *et al*. (2009) recorded 13 distinct VSG transcripts in metacyclic cells, with the most abundant comprising 24% of the total number, and 26 distinct VSG transcripts in bloodstream forms, with the most abundant contributing 62% of the total [12]. This supports the established experimental model in which most individuals of a *T*. *congolense* population express the same active VSG, while a few individuals express a range of low abundance alternatives. In fact, when combined, the 12 least abundant VSG EST were only 0.5% of all VSG transcripts in bloodstream forms [12]. Similarly in *T*. *brucei*, Jensen et al. (2009) identified in a microarray-based study cohorts of less abundant transcripts in addition to the known, active VSG, some of which were expressed most abundantly in the insect stages [16]. In contrast, a previous analysis of VSG transcripts in *T*. *vivax* using 454 sequencing technology concluded that only one VSG was expressed [14].

Proteomic analyses have presented a similar picture. In *T*. *congolense*, 11 different VSGs were identified across all life stages [13]. Four were confidently associated with the metacyclic stage while two others were significantly enriched in bloodstream forms (including the known, active VSG). In proteomic comparisons of procyclic and bloodstream forms of *T*. *brucei*, one proteome identified 10 canonical VSGs [22], while another only two [21], although different *T*. *brucei* strains were used. These did not include the active VSG because neither study used the reference strain (927), and so the active VSG did not map to a VSG gene in the reference genome. Consequently, the 10 VSGs identified by Butter et al. (2013) are all low abundance alternatives, represented by few peptides (< 7) and achieving poor coverage (< 9%) [22]. Three of these low abundance VSGs were preferentially expressed in procyclic forms [22]. Taking these previous data together, their obvious methodological variations notwithstanding, low abundance alternatives to the dominant VSG are observed at both the transcript and protein levels in both *T*. *brucei* and *T*. *congolense*. The role, if any, of these ‘accessory’ VSGs is unclear; some are very likely metacyclic VSGs and it is known that expression of these can continue several days after transmission [45] and so could be present in bloodstream forms. Alternatively, ‘accessory’ VSGs may simply be rare antigens expressed by low frequency subpopulations, or produced by inefficiency in the mechanism silencing inactive VSG expression sites.

In contrast to the previous result [14], we observed several low abundance VSGs in *T*. *vivax* consistent with the expression profiles of VSG observed in *T*. *brucei* and *T*. *congolense*. The two dominant VSG sequences were superabundant at both transcript and peptide levels. Therefore the identity of the active VSG remained constant in the period between RNA and protein preparation, meaning that this is unlikely to represent a transition between two VSGs and that *T*. *vivax* strain IL1392 probably a mixture of parasites expressing one of two different VSGs. Both of these active VSG belong to Fam24, the subtype homologous to canonical b-type VSG in *T*. *brucei* and *T*. *congolense* [11]. In a similar fashion to *T*. *congolense*, the less abundant VSG in *T*. *vivax* may represent metacyclic VSGs. One VSG, TvY486_0027560, may be a metacyclic VSG in this strain as it was preferentially expressed in the metacyclic form (its transcript was not recorded). Finally, two VSG-like sequences belonging to Fam25, a *T*. *vivax*-specific subtype [11], are preferentially expressed at low levels in epimastigotes. The roles of Fam25 and 26 genes remain mysterious, and there is no definitive evidence that they encode variant antigens.

**Fig 5:**
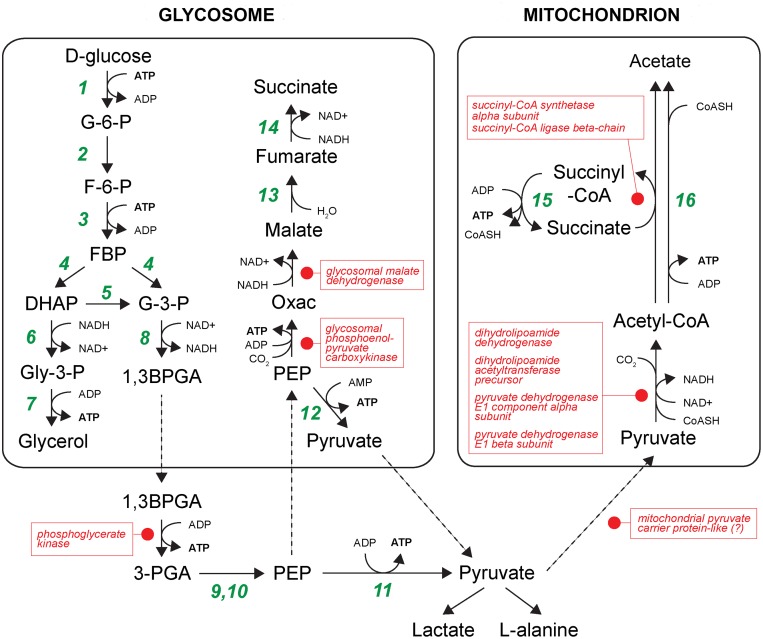
Energy metabolism in African trypanosomes, noting the position of enzymes with *T*. *vivax*-specific developmental regulation. Glycolysis takes place within a specialized organelle, the glycosome, after which further substrate level phosphorylation takes place through the conversion of phosphoenolpyruvate ultimately to succinate in the glycosome, and through the conversion of pyruvate into acetate in the mitochondrion. Points marked with red dots and labels shaded red refer to proteins that are preferentially expressed during the vertebrate stage of *T*. *vivax* but in the insect stages of *T*. *brucei* (after Besteiro *et al*. 2005). Note that we could not differentiate between cytosolic and glycosomal phosophoglycerate kinase isoforms using our proteomic data. Abbreviations: 1,3BPGA, 1,3-bisphosphoglycerate; CoASH, coenzyme A; DHAP, dihydroxyacetone phosphate; F-6-P, fructose 6-phosphate; FBP, fructose 1,6-bisphosphate; G-3-P, glyceraldehyde 3-phosphate; G-6-P, glucose 6-phosphate; GLU, glutamate; Gly-3-P, glycerol 3-phosphate; Oxac, oxaloacetate; PEP,phosphoenolpyruvate; 3-PGA, 3-phosphoglycerate; SucCoA, succinyl-CoA. Enzymes are: 1) hexokinase: 2) glucose-6-phosphate isomerase; 3) phosphofructokinase; 4) aldolase; 5) triose-phosphate isomerase; 6) glycerol-3-phosphate dehydrogenase; 7) glycerol kinase; 8) glyceraldehyde-3-phosphate dehydrogenase; 9) phosphoglycerate mutase; 10) enolase; 11) pyruvate kinase; 12) pyruvate phosphate dikinase; 13) glycosomal fumarase; 14) NADH-dependent fumarate reductase; 15) acetate:succinate CoA-transferase; 16) possibly acetyl-CoA synthetase.

Beyond the differences in genetic repertoire that are evident from comparative genomics, it is presumed that differences in the regulation of conserved genes will contribute to phenotypic differences between African trypanosomes. The cohort of conserved genes identified in Figs 2 and 3 that are regulated conversely in *T*. *vivax* relative to *T*. *brucei* (and probably *T*. *congolense*) indicate that this is so. In the bloodstream stage, African trypanosomes exclusively employ glycolysis to exploit abundant glucose in host plasma to generate ATP via substrate-level phosphorylation in the glycosome [46]. In the tsetse fly, where glucose is limited but amino acids such as proline are present in the host hemolymph, the parasites generate ATP through gluconeogenesis and oxidative phosphorylation in the mitochondrion [46]. In this regard, *T*. *vivax* is consistent; all glycolytic enzymes are preferentially expressed in the bloodstream form, where they are among the most abundant transcripts and peptides, and all components of the electron transfer chain are preferentially expressed in the epimastigote (S3 and S4 Tables). The species differences highlighted in Fig 3 concern the metabolic steps linking glycolysis and events in the mitochondrion, i.e. pyruvate metabolism (Fig 5).

Experimental evidence indicates that *T*. *brucei* produces ATP during its insect stage by further substrate-level phosphorylation in the glycosome, by catabolizing phosphoenolpyruvate (PEP), and in the mitochondrion by catabolizing pyruvate. This results in insect forms excreting succinate and acetate, while bloodstream forms excrete pyruvate [47]. Accordingly, the enzymes for converting PEP into succinate and pyruvate into acetate are preferentially expressed in the procyclic form of *T*. *brucei* [21, 22]. Fig 5 describes the points in this pathway where differential expression is reversed in *T*. *vivax*. We see that enzymes for the catabolism of PEP, such as glycosomal malate dehydrogenase and glycosomal phosphoenolpyruvate carboxykinase, and for the conversion of pyruvate to acetate, i.e. multiple components of the pyruvate dehydrogenase complex and of the succinyl-CoA synthetase complex, are significantly more active in the bloodstream form than in the epimastigote. Additionally, the fumarase responsible for reaction 13 in Fig 5, while upregulated in procyclic form *T*. *brucei*, is constitutively expressed in *T*. *vivax* (TvY486_1105200; FC = 0.04). However, the final enzyme in the pathway (NADH-dependent fumarate reductase; reaction 14) is preferentially expressed in the insect stages in both species. We speculate that some other genes in Table 1 support this function; for example, TvY486_0901260, which possesses a mitochondrial pyruvate carrier protein domain homologous to mt1 in Humans, which is required to import pyruvate across the inner mitochondrial membrane [48], and TvY486_0702860, which encodes a bacterial-type nitro-FMN oxidoreductase that might serve to regenerate NAD+ [49].

Thus, we would predict that *T*. *vivax* excretes fumarate, acetate and perhaps succinate in its bloodstream stage rather than in the insect. It is not clear why *T*. *vivax* would benefit from pyruvate metabolism in the bloodstream when substrate-level phosphorylation using glucose should suffice. However, in the insect stage, when the parasite remains in the proboscis and without access to the hemolymph, it could be that such metabolism serves little purpose. Therefore, this may reflect a lack of upregulation in the epimastigote rather than adaptive upregulation in the bloodstream form, illustrating how life cycle variation has affected the regulation of energy metabolism in these organisms.

The first global perspective on gene expression in *T*. *vivax* has confirmed that a broadly similar process of developmental regulation occurs in all African trypanosome species. However, subtle differences, for instance in energy metabolism and putative cell surface molecules, offer new insights into the molecular basis for the life cycle differences that exist between species. Beyond the background of conservation, this study has confirmed the presence of numerous *T*. *vivax*-specific gene families and shown that these are developmentally regulated, indicating that the surface of *T*. *vivax* differs quite substantially from the model derived from other African trypanosomes.

## Supporting Information

S1 Fig Exemplar correlations of log-transformed transcript abundance (log2 FPKM), as estimated by Cufflinks, between replicate analyses of bloodstream form (BSF), metacyclic-stage (MET) and epimastigote (EPI) parasites. (DOCX)

S2 Fig Multi-dimensional scaling plot of replicate RNA-seq data, as produced by edgeR [34]. Leading log-fold change in the first dimension is plotted on the x-axis and the second dimension is on the y-axis. Distances here correspond to leading log-fold-changes between replicates in each pairwise comparison of life stages: BSF vs EPI (left), BSF vs MET (centre) and EPI vs MET (right). These plots demonstrate that replicates cluster by life stage, reflecting the consistency in transcript abundance among replicates of the same stage.
(DOCX)

S3 Fig Principle component analysis plot of protein abundance in all *T*. *vivax* life stages, produced using Progenesis. Normalized protein abundance levels across different samples were plotted to determine the principle axes of abundance variation. The first principle component is plotted on the x-axis and the second is plotted on the y-axis. The mass of grey numbers in the background refer to individual data points (proteins). Data points derived from individual replicates collected in each life stage are summed and represented by coloured dots: red (BSF), green (MET) and blue (EPI). The coloured dots cluster by life stage reflecting the consistency in expression profile provided by replicates of each condition.
(DOCX)

S4 Fig Scatterplots showing the correlation of log-transformed transcript abundance (log2 FPKM; y-axis) and protein abundance (x-axis) estimates in pairwise comparisons of *T*. *vivax* life stages for all observed proteins (a-c) and for those displaying significant differential expression only (d-f). The number of comparisons possible for each life stage comparison is given in brackets.
(DOCX)

S1 Table Transcript abundance values in Fragments Per Kilobase Mapped (FPKM) for all Trypanosoma vivax Y486 genes across the three developmental life stages (bloodstream-form (BSF), metacyclic (MET), epimastigote (EPI)). Genes without a non-zero value in all three conditions have been removed.
(XLSX)

S2 Table Peptide abundance values as calculated by Progenesis for detected Trypanosoma vivax Y486 predicted proteins across the three developmental life stages (bloodstream-form (BSF), metacyclic (MET), epimastigote (EPI)). (XLSX)

S3 Table Transcripts showing significant differential expression in pairwise comparisons of life cycle stage. Significance is defined as p < 0.05 and FC ≥ 2.
(XLSX)

S4 Table Predicted proteins showing significant differential expression in pairwise comparisons of life cycle stage. Significance is defined as q < 0.05 and FC ≥ 2.
(XLSX)
