# Supplementary material for: Global Gene Expression Profiling through the Complete Life Cycle of Trypanosoma vivax
Source: PLoS Negl Trop Dis. 2015 Aug 12;9(8):e0003975. doi: 10.1371/journal.pntd.0003975 (PMC4534299; doi:10.1371/journal.pntd.0003975)
Supplement: S1 Fig — (DOCX) [file pntd.0003975.s001.docx]

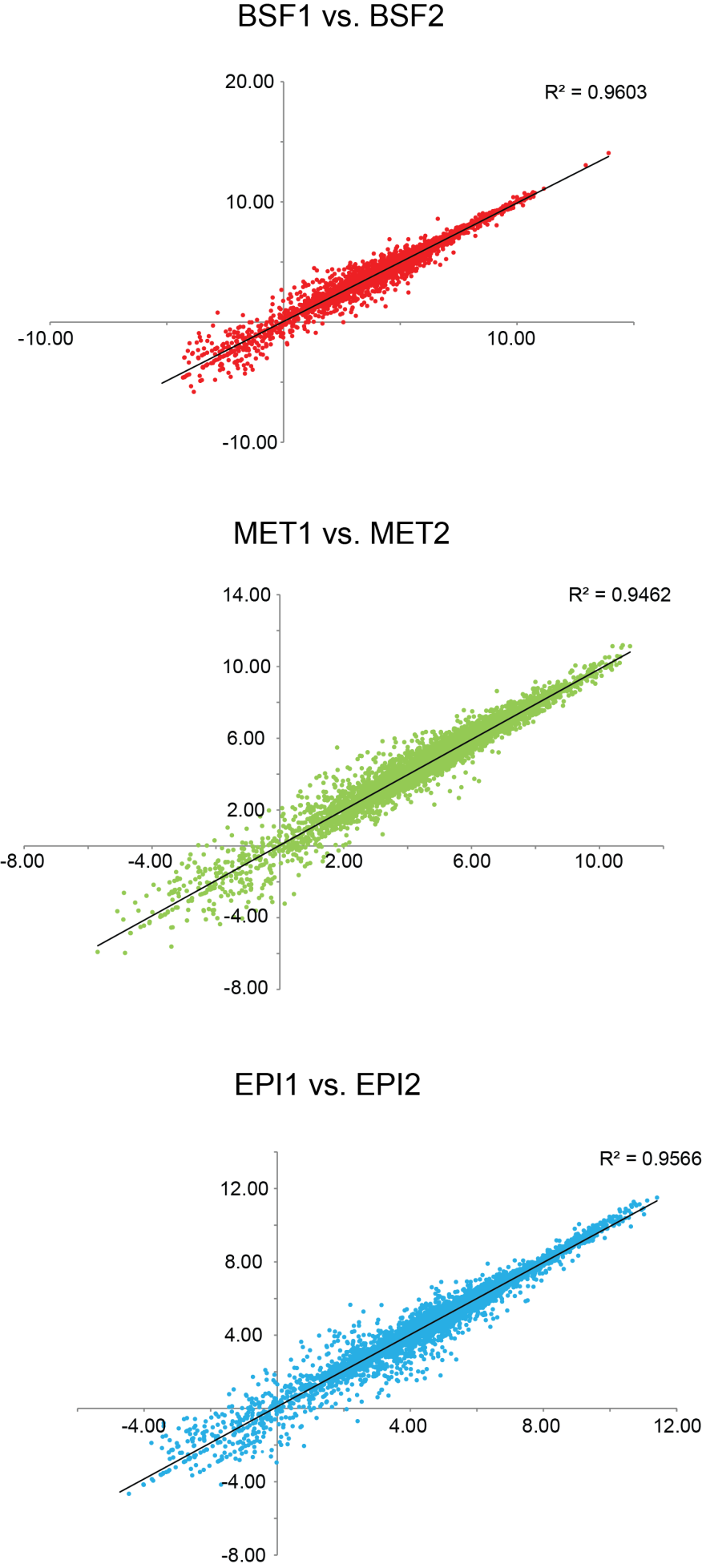


**Supplementary Figure 1.** Exemplar correlations of log-transformed transcript abundance (log2 FPKM), as estimated by Cufflinks, between replicate analyses of bloodstream form (BSF), metacyclic-stage (MET) and epimastigote (EPI) parasites.
