# Supplementary material for: Global Gene Expression Profiling through the Complete Life Cycle of Trypanosoma vivax
Source: PLoS Negl Trop Dis. 2015 Aug 12;9(8):e0003975. doi: 10.1371/journal.pntd.0003975 (PMC4534299; doi:10.1371/journal.pntd.0003975)
Supplement: S2 Fig — Leading log-fold change in the first dimension is plotted on the x-axis and the second dimension is on the y-axis. Distances here correspond to leading log-fold-changes between replicates in each pairwise comparison of life stages: BSF vs EPI (left), BSF vs MET (centre) and EPI vs MET (right). These plots demonstrate that replicates cluster by life stage, reflecting the consistency in transcript abundance among replicates of the same stage. (DOCX) [file pntd.0003975.s002.docx]

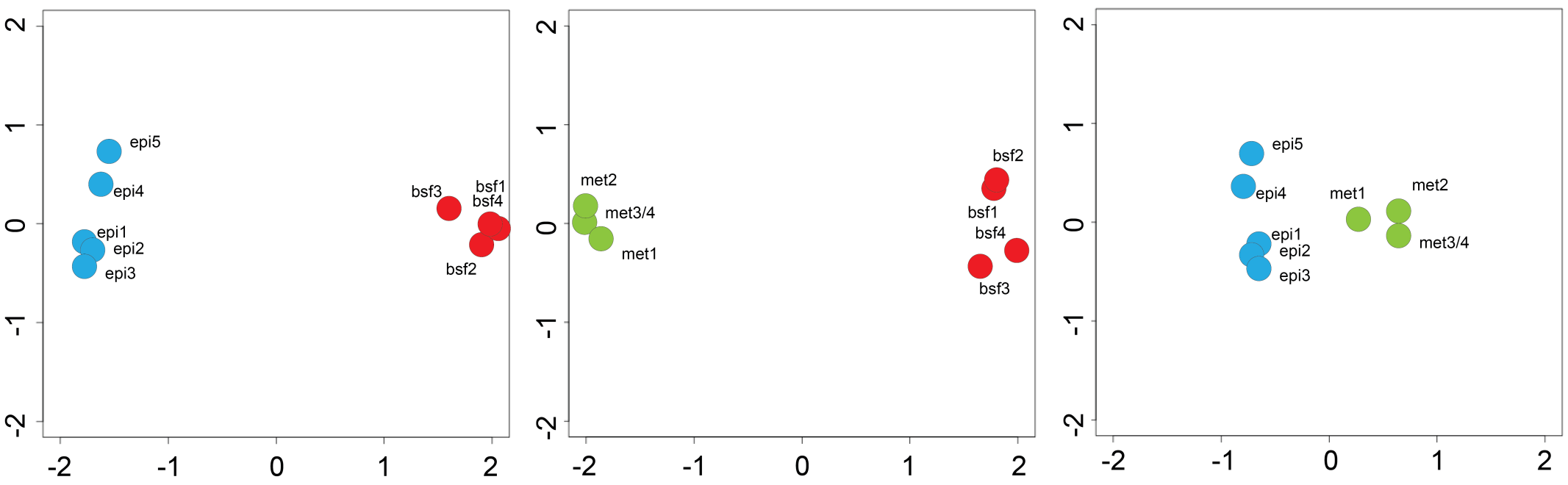


**Supplementary Figure 2**. Multi-dimensional scaling plot of replicate RNA-seq data, as produced by edgeR [34]. Leading log-fold change in the first dimension is plotted on the x-axis and the second dimension is on the y-axis. Distances here correspond to leading log-fold-changes between replicates in each pairwise comparison of life stages: BSF vs EPI (left), BSF vs MET (centre) and EPI vs MET (right). These plots demonstrate that replicates cluster by life stage, reflecting the consistency in transcript abundance among replicates of the same stage.
