# Supplementary material for: Global Gene Expression Profiling through the Complete Life Cycle of Trypanosoma vivax
Source: PLoS Negl Trop Dis. 2015 Aug 12;9(8):e0003975. doi: 10.1371/journal.pntd.0003975 (PMC4534299; doi:10.1371/journal.pntd.0003975)
Supplement: S3 Fig — Normalized protein abundance levels across different samples were plotted to determine the principle axes of abundance variation. The first principle component is plotted on the x-axis and the second is plotted on the y-axis. The mass of grey numbers in the background refer to individual data points (proteins). Data points derived from individual replicates collected in each life stage are summed and represented by coloured dots: red (BSF), green (MET) and blue (EPI). The coloured dots cluster by life stage reflecting the consistency in expression profile provided by replicates of each condition. (DOCX) [file pntd.0003975.s003.docx]

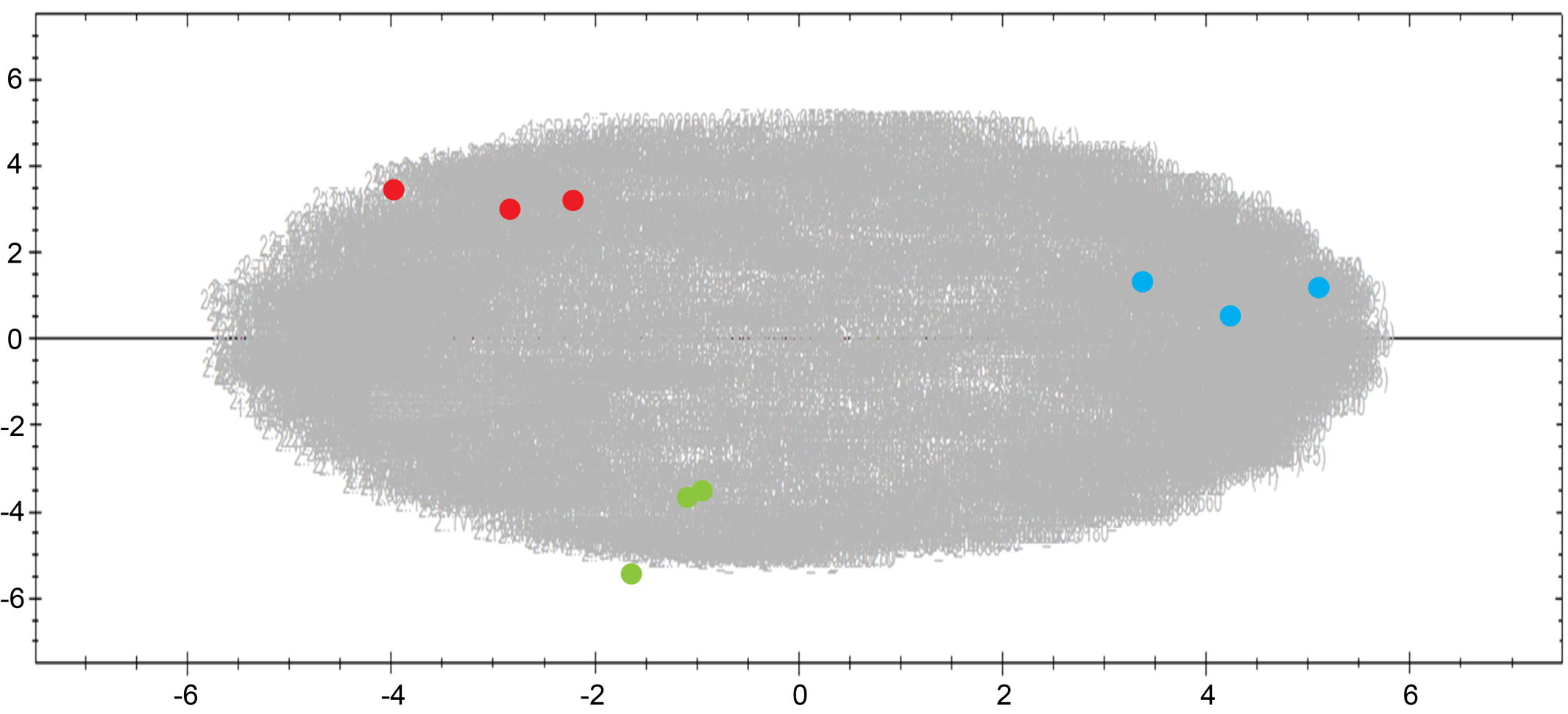


**Supplementary Figure 3.** Principle component analysis plot of protein abundance in all *T. vivax* life stages, produced using Progenesis. Normalized protein abundance levels across different samples were plotted to determine the principle axes of abundance variation. The first principle component is plotted on the x-axis and the second is plotted on the y-axis. The mass of grey numbers in the background refer to individual data points (proteins). Data points derived from individual replicates collected in each life stage are summed and represented by coloured dots: red (BSF), green (MET) and blue (EPI). The coloured dots cluster by life stage reflecting the consistency in expression profile provided by replicates of each condition.
