# Supplementary material for: Global Gene Expression Profiling through the Complete Life Cycle of Trypanosoma vivax
Source: PLoS Negl Trop Dis. 2015 Aug 12;9(8):e0003975. doi: 10.1371/journal.pntd.0003975 (PMC4534299; doi:10.1371/journal.pntd.0003975)
Supplement: S4 Fig — The number of comparisons possible for each life stage comparison is given in brackets. (DOCX) [file pntd.0003975.s004.docx]

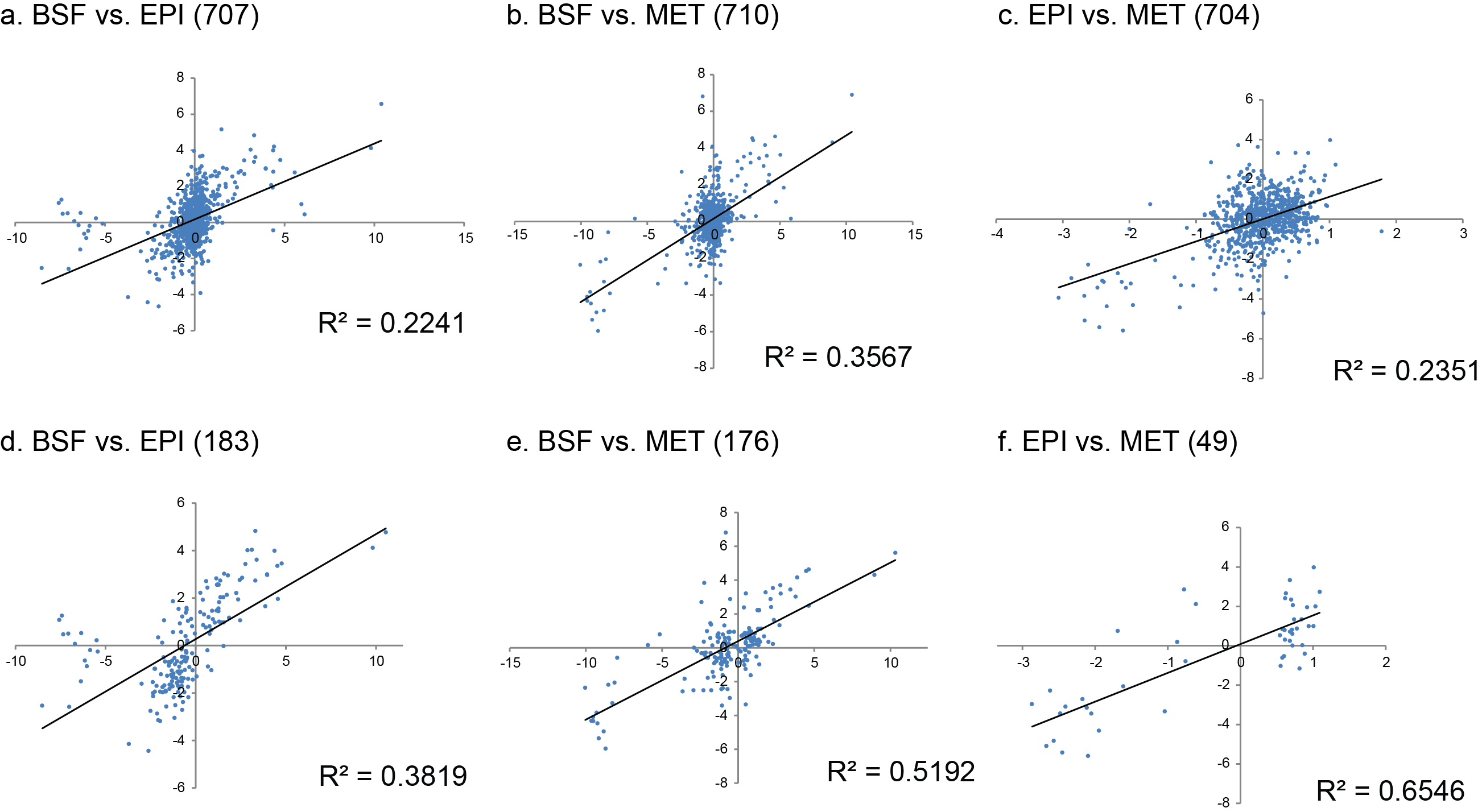


**Supplementary Figure 4.** Scatterplots showing the correlation of log-transformed transcript abundance (log2 FPKM; y-axis) and protein abundance (x-axis) estimates in pairwise comparisons of *T. vivax* life stages for all observed proteins (**a-c**) and for those displaying significant differential expression only (**d-f**). The number of comparisons possible for each life stage comparison is given in brackets.
